# Primary and secondary functions of HLA-E are determined by stability and conformation of the peptide-bound complexes

**DOI:** 10.1016/j.celrep.2022.110959

**Published:** 2022-06-14

**Authors:** Lucy C. Walters, Daniel Rozbesky, Karl Harlos, Max Quastel, Hong Sun, Sebastian Springer, Robert P. Rambo, Fiyaz Mohammed, E. Yvonne Jones, Andrew J. McMichael, Geraldine M. Gillespie

**Affiliations:** 1 Nuffield Department of Medicine Research Building, Nuffield Department of Medicine, University of Oxford, Roosevelt Drive, Oxford OX3 7FZ, UK; 2 Department of Cell Biology, Faculty of Science, Charles University, Prague, Czech Republic; 3 Division of Structural Biology, Wellcome Centre for Human Genetics, University of Oxford, Roosevelt Drive, Oxford OX3 7BN, UK; 4 Department of Laboratory Medicine, The First Affiliated Hospital, China Medical University, Shenyang, China; 5 Department of Life Sciences and Chemistry, Jacobs University Bremen, Bremen, Germany; 6 Diamond Light Source, Harwell Science and Innovation Campus, Didcot, Oxfordshire OX11 0DE, UK; 7 Institute of Immunology and Immunotherapy, University of Birmingham, Edgbaston, Birmingham B15 2TT, UK

**Keywords:** MHC-E, HLA-E, small-angle X-ray scatter, SAXS, X-ray crystallography, VL9, MHC Ia, NK cells, NKG2A, CD8 T cells, T cell receptor

## Abstract

MHC-E regulates NK cells by displaying MHC class Ia signal peptides (VL9) to NKG2A:CD94 receptors. MHC-E can also present sequence-diverse, lower-affinity, pathogen-derived peptides to T cell receptors (TCRs) on CD8^+^ T cells. To understand these affinity differences, human MHC-E (HLA-E)-VL9 versus pathogen-derived peptide structures are compared. Small-angle X-ray scatter (SAXS) measures biophysical parameters in solution, allowing comparison with crystal structures. For HLA-E-VL9, there is concordance between SAXS and crystal parameters. In contrast, HLA-E-bound pathogen-derived peptides produce larger SAXS dimensions that reduce to their crystallographic dimensions only when excess peptide is supplied. Further crystallographic analysis demonstrates three amino acids, exclusive to MHC-E, that not only position VL9 close to the α2 helix, but also allow non-VL9 peptide binding with re-configuration of a key TCR-interacting α2 region. Thus, non-VL9-bound peptides introduce an alternative peptide-binding motif and surface recognition landscape, providing a likely basis for VL9- and non-VL9-HLA-E immune discrimination.

## Highlights

• Solution-based X-ray scattering analysis of HLA-E-bound VL9 reveals compact folded forms
• HLA-E bound to pathogen-derived epitopes form heterogeneous ensembles in solution
• Disrupted E pocket occupancy characterizes HLA-E-bound pathogen-derived peptides
• An HLA-E α2 reconfiguration forms a shared immune landscape for non-VL9 peptides

## Graphical abstract

**Figure:**
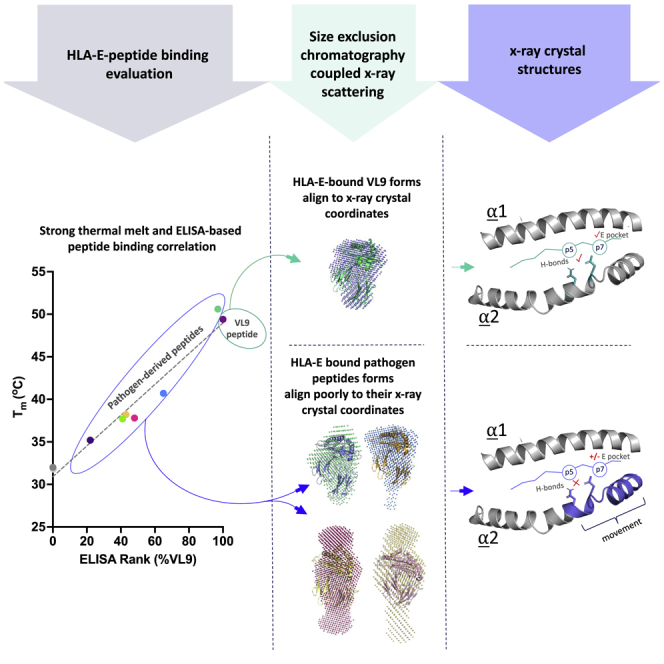

## Teaser

Using small-angle X-ray scattering, Walters et al. report that HLA-E in complex with pathogen-derived peptides form heterogeneous protein ensembles in-solution. X-ray structural analysis reveals that disrupted peptide interactions involving the E pocket plus α2 helical residue are shared among these HLA-E-peptides complexes, creating antigen-recognition surfaces that diverge from optimally bound HLA-E-VL9.

## Introduction

The primary function of major histocompatibility complex E (MHC-E), evolutionally preserved in humans, primates, and rodents, is to present a short peptide from the signal sequence of classical MHC Ia molecules (VL9) to NKG2x-CD94 receptors expressed on natural killer (NK) cell and specific CD8 T cell populations. Cell surface-expressed HLA-E-VL9 signify MHC Ia presentation pathway integrity, regulating NK cell-mediated lysis through CD94/NKG2x receptor engagement by balancing cellular inhibition (CD94/NKG2A) and activation (CD94/NKG2C) (Borrego et al., 1998; Braud et al., 1997, 1998; Kaiser et al., 2005; Lee et al., 1998b). Yet, in specific circumstances, MHC-E can function like classical MHC Ia by presenting antigenic self and foreign peptides that are recognized by T cell receptors expressed on CD8^+^ T cells (Hansen et al., 2016; Joosten et al., 2010; McMurtrey et al., 2017). The recent description of vaccine-induced MHC-E-restricted CD8^+^ T cells specific for simian immunodeficiency virus (SIV) that completely eradicate early viral challenge in monkeys has awakened interest in such T cells (Hansen et al., 2016; Malouli et al., 2021). Mycobacteria-specific HLA-E-restricted CD8^+^ T cells have also been identified in humans (Joosten et al., 2010; McMurtrey et al., 2017; Prezzemolo et al., 2018).

The unprecedented allelic polymorphism of MHC Ia molecules allows broad sampling of diverse pathogen-derived peptides at the population level. By contrast, balancing selection at the dimorphic HLA-E (human MHC-E) locus maintains similar frequencies of the two non-synonymous allotypes, HLA-E^∗^01:01 and HLA-E^∗^01:03, in ethnically diverse populations (Geraghty et al., 1992; Grimsley and Ober, 1997). These allotypes differ by a single amino acid substitution (R to G) at position 107 outside the peptide-binding groove (PBG), resulting in overlapping peptide-binding repertoires with no detectable difference in VL9 presentation (O’Callaghan et al., 1998; Strong et al., 2003). Such limited polymorphism implies that conserved presentation of VL9 to NK cell receptors is the primary function of HLA-E. However, the minimal allelic polymorphism and shared peptide-binding repertoires position HLA-E as a particularly attractive “universal” restriction element for T-cell-targeted vaccination and therapeutic strategies.

HLA-E was originally shown to bind a highly conserved set of nonameric VL9 peptides (VMAPRT[V/L][L/V/I/F]L) comprising residues 3–11 of MHC Ia (HLA-A/B/C/G) leader sequences, with the exception of HLA-E and HLA-F due to N-terminal leader sequence truncations (Braud et al., 1997; Lee et al., 1998a). Five VL9 peptide side chains occupy pockets in the HLA-E PBG; primary anchor positions 2 (M) and 9 (L) sit in the B and F pockets, respectively, and three secondary residues at position 3 (A), 6 (T), and 7 (L), respectively, occupy the D, C, and E pockets (Hoare et al., 2008; O’Callaghan et al., 1998; Strong et al., 2003). Consistent with a limited peptide repertoire, in part due to the requirement to occupy an unusually large number of pockets, VL9 peptides dominate the HLA-E-presented ligandome (McMurtrey et al., 2017). In contrast, remarkable sequence diversity marks MHC-E-bound peptides that stimulate CD8^+^ T cells (Hansen et al., 2016; McMurtrey et al., 2017; Prezzemolo et al., 2018; Vance et al., 1998; Wu et al., 2018). No sequence motif encompasses these epitopes, and the majority demonstrate negligible HLA-E binding (<10% of VL9 binding) in a sandwich ELISA-based screen that employs a conformation-specific, anti-HLA-E capture antibody combined with anti-β2m detection (Walters et al., 2020). However, this assay identified pathogen-derived epitopes that demonstrate “intermediate” HLA-E binding (normalized signals >20% of VL9); these show a sequence motif and include HIV-, SIV-, Mtb-, and EBV-derived peptides (Walters et al., 2018).

Here, we characterized the biophysical and structural properties of HLA-E^∗^01:03 in complexes with intermediate-affinity pathogen-derived epitopes, comparing their structures with HLA-E-bound VL9. We uncovered striking differences in the conformational ensemble of VL9 versus pathogen-derived peptide-HLA-E complexes by using small-angle X-ray scattering (SAXS) to explore their conformation in solution, finding congruity between SAXS data, thermal melting temperatures, and ELISA-based peptide-binding data. Furthermore, structural determination of HLA-E in complex with the same epitopes highlighted the importance of E pocket occupancy for complex stability, providing a molecular rationale for the differential biophysical properties observed. Finally, structural analyses revealed an altered configuration in the HLA-E α2 helical kink region primarily involving E152, conserved among structures of pathogen-derived peptide-bound HLA-E but absent in published HLA-E-VL9 structures. Our data suggest that distinctive structural features and biophysical properties contribute to HLA-E discrimination in steady (VL9-bound) versus pathological (pathogen peptide-bound) state, respectively. These structural differences go some way to explain how VL9 dominates the HLA-E peptidome to maintain regulatory control by NK cells, the primary biological function of HLA-E. Our findings also illustrate how the antigenic surface distinguishing non-VL9 bound from VL9-loaded HLA-E complexes generates an immune recognition footprint more generally shared among pathogen-bound epitopes.

## Results

### Distinct blue native gel signatures for HLA-E-VL9 versus pathogen epitope-bound HLA-E

Six pathogen-derived HLA-E-restricted epitopes that demonstrated HLA-E-binding capacity of 20%–100% of the positive control VL9 peptide signal in our sandwich ELISA-based peptide-binding assay were evaluated by blue native gel (BNG) analysis (Figure S1) (Walters et al., 2018). Peptide exchange reactions were conducted using HLA-E complexes pre-refolded with a UV-sensitive VL9 variant (VMAPJTVLL) incubated with 12 M molar excess test peptide prior to BNG sampling. Differential gel signatures were observed for the control VL9 leader peptide relative to the pathogen-derived peptides IL9, Mtb14, RL9HIV, RL9SIV, and BZLF1. Whereas HLA-E-VL9 generated a single compact gel band, most pathogen-derived peptides gave diffuse gel band forms. Two band forms were visible for BZLF1, including both a faint, compact band and a dominant, diffuse form. Mtb44, the only pathogen-derived peptide to demonstrate comparable binding to VL9 (Walters et al., 2018), generated a compact gel band. These data suggested that HLA-E refolded with these peptides, despite generating single peaks on size exclusion chromatography, might not be homogeneous.

**Figure 1:**
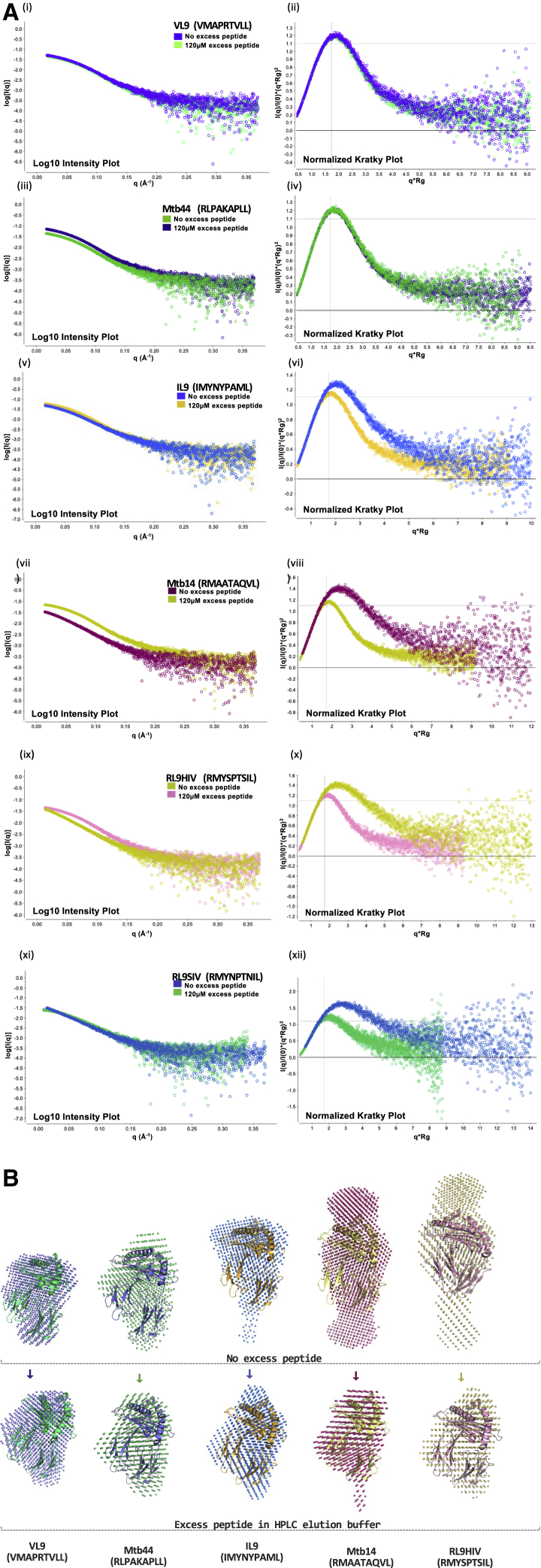
SEC-SAXS analysis of peptide-HLA-E complexes (A) (i), (iii), (v), (vii), (ix), and (xi): Log_10_ scattering intensity plots for HLA-E SEC-SAXS experiments. Scattering intensity curves for HLA-E refolds run in the absence or presence of 120 μM excess peptide during elution are plotted. On the x axis is the scattering vector q (Å^−1^) versus the scattered intensity, I(q), log-scale, on the y axis. (ii), (iv), (vi), (viii), (x), and (xii): Normalized Kratky plots with superimposed curves corresponding to HLA-E SEC-SAXS experiments with or without 120 μM excess peptide. Modulated Gaussian curves are color-coded according to legends in adjacent log_10_ intensity plots. The scattering vector multiplied by the radius of gyration is plotted on the x axis versus the scattering intensity I(q) divided by the experiment’s I(0) multiplied by (q^∗^R_g_)^2^ on the y axis. (B) *Ab initio* DAMMIF models (small dots) represent the average conformational protein state in solution. Top: SEC-SAXS runs where HLA-E complexes were injected onto the HPLC column in the presence of 120 μM excess peptide but without excess peptide during elution. Bottom: 120 μM excess peptide was present throughout injection and elution. HLA-E-peptide structural coordinates superimposed via SUPCOMB onto their peptide-equivalent SEC-SAXS-based bead models are reported for VL9 (PDB: 1MHE), Mtb44 HLA-Mtb44 (PDB: 6GH1), IL9 (structure reported here), Mtb14 (structure reported here) and RL9HIV (PDB: 6LH1).

### SEC-SAXS reveals peptide-dependent differences in the HLA-E conformational ensemble

We next used size exclusion chromatography-coupled small-angle X-ray scattering (SEC-SAXS) to probe the protein-conformational ensemble for VL9 versus pathogen peptide-bound HLA-E. Scattering data from X-ray-exposed refolded HLA-E^∗^01:03 protein samples were collected during high-performance liquid chromatography (HPLC) elution, from which the radius of gyration (R_g_) and maximum dimension (d_max_) of protein complexes in solution were calculated, following subtraction of buffer-induced scattering. DAMMIF *ab initio* models were also generated which represent the average HLA-E conformer in solution (Franke and Svergun, 2009; Volkov and Svergun, 2003).

The resolution limits of SAXS are inferior to X-ray crystallography. However, unlike the static, lattice-restrained snapshot afforded by X-ray crystallography, SAXS can detect dynamic protein-folded states and large conformational adjustments in solution (Kikhney and Svergun, 2015). Consistent with the BNG results, we observed pronounced differences in alignment of DAMMIF models to HLA-E crystallographic coordinates for VL9 (VMAPRTVLL)-bound HLA-E versus HLA-E refolded with the pathogen-derived epitope peptides RL9HIV (RMYSPTSIL), RL9SIV (RMYNPTNIL), IL9 (IMYNYPAML), Mtb14 (RMAATAQVL), and BZLF1 (SQAPLPCVL) (Figures 1 and S2). Whereas superposition of the HLA-E-VL9 crystal structure to SEC-SAXS-derived DAMMIF models revealed similar global dimensions for VL9-refolded HLA-E (O’Callaghan et al., 1998), poor fits were observed for HLA-E refolded with the pathogen-derived peptides. The model was consistent with the higher d_max_ and R_g_ values implying that these epitopes do not support uniformly compact, fully folded HLA-E complex forms in solution. This finding was unexpected, as crystal structures of HLA-E in complex with RL9HIV, IL9, and Mtb14 exhibited comparable global dimensions and superposed to structures of HLA-E-VL9 (Walters et al., 2018).

Only the Mtb44 (RLPAKAPLL) peptide, which generated comparable ELISA-based peptide-binding signals and BNG signatures to VL9, produced a compact DAMMIF model with strong alignment to structural coordinates and comparable R_g_ and D_max_ values to HLA-E-VL9 in SEC-SAXS analyses (Figure 1 and Table 1). Finally, a number of highly immunogenic HLA-E-restricted epitopes that exhibited weak or non-binding in the ELISA-based method, including the immunodominant SIV epitope, EK9 (EKQRESREK) (Hansen et al., 2016; Walters et al., 2020), produced proportionally smaller protein peaks, thus yielding insufficient X-ray scattering for reliable downstream data processing.

**Table 1:** SEC-SAXS-derived datasets for peptide-HLA-E complexes collected with and without 120 μM excess peptide

Peptide ID	Sequence	Organism	Excess peptide (μM)	Rg (Å)	D_max_ (Å)	Molecular envelope	Molecular envelope
						Vol (Å3)	Vol %change
VL9	VMAPRTVLL	Human	0	26.4	87	78,628	+4
			120	26.1	90	82,078	
Mtb44	RLPAKAPLL	Mtb	0	26.6	97	84,171	+1
			120	26.4	93	84,996	
IL9	IMYNYPAML	Mtb	0	28.8	93	85,348	−2
			120	25.4	91	83,316	
Mtb14	RMAATAQVL	Mtb	0	35.9	120	123,647	−32
			120	25.7	99	83,670	
RL9HIV	RMYSPTSIL	HIV	0	38.8	127	146,874	−42
			120	26.7	91	85,230	
RL9SIV	RMYNPTNIL	SIV	0	42.6	155	161,392	−34
			120	29.1	104	107,137	

Rows represent SEC-SAXS experiments conducted with or without 120 μM excess peptide in HPLC elution buffers. The radius of gyration (R_g_), maximum dimension (d_max_) (both Å), SEC-SAXS “dot” models (Å^3^), and % change in model volume following excess peptide addition are specified.

SEC-SAXS analyses also revealed conformational homogeneity for HLA-E-VL9 refolds, as evidenced by similar Rg values across X-ray-exposed frames of the HPLC-eluted protein peaks (Figure S3A). However, non-constant and decreasing Rg values across the eluted protein peak were observed for HLA-E refolded with peptides RL9HIV, RL9SIV, Mtb14, IL9, and BZLF1. Accordingly, stratification of SEC-SAXS HPLC peaks into leading and tailing fractions prior to downstream processing confirmed sample heterogeneity, as indicated by discrepancies in R_g_ or d_max_ values and differentially positioned modulated Gaussian curve peaks on normalized Kratky plots between peak fractions (Figures S3B and S3C). Together, the heterogeneous conformational protein ensembles and poor alignment of DAMMIF models to HLA-E crystallographic coordinates imply that the majority of intermediate strength HLA-E-restricted pathogen-derived epitopes drive suboptimal HLA-E complex formation despite their previously reported immunogenicity *in vivo* (Hansen et al., 2016; McMurtrey et al., 2017; van Meijgaarden et al., 2015).

To test whether this phenomenon was related to peptide-binding affinity, we performed SEC-SAXS in the continued presence of excess peptide. Notably, molar excess peptide added to the HPLC elution buffer transformed poorly aligned DAMMIF models of pathogen epitope-refolded HLA-E (RL9HIV, RL9SIV, IL9, and Mtb14) into compact forms with strong alignment to published HLA-E crystal structures (O’Callaghan et al., 1998; Walters et al., 2018) and similar to that observed for VL9-refolded HLA-E (Figure 1B). Additionally, superimposed log_10_ intensity plots, which show the intensity of scattered X-rays over the small-angle range, revealed differentially shaped curves in the absence or presence of excess peptide (Figure 1A). Dimensionless Kratky plots normalized for particle mass and concentration also demonstrated global conformational discrepancies between protein populations eluted with and without excess peptide. The normalized Kratky plot units were chosen such that the peak of the modulated Gaussian curve corresponded to q^∗^Rg = √3 with a magnitude of 3·e−1, regardless of protein size and concentration when the Guinier’s approximation is obeyed for globular and compact proteins. A shift from this peak position signifies deviation from the Guinier’s approximation, indicating an increased degree of protein flexibility or conformational asymmetry. For HLA-E, the peak of the modulated Gaussian curve shifted further toward q^∗^R_g_ = √3 (x axis) with a magnitude of 3·e−1 (y axis) in the presence of excess Mtb14, RL9HIV, RL9SIV, or IL9 peptide, consistent with more compact, globular protein states (Figures 1A, vi, viii, x, and xii). By contrast, greater deviations from this specified Gaussian curve maximum for corresponding HLA-E refolds in the absence of excess peptide during elution indicate a greater degree of protein flexibility or conformational asymmetry. HLA-E refolds with the higher-affinity VL9 and Mtb44 peptides were less impacted by excess peptide addition, as evidenced by stronger alignment in the superimposed scattering intensity curves and modulated Gaussian curves (Figures 1A, i–iv). So, although all pathogen epitope-HLA-E samples were injected onto the HPLC column in the presence of excess peptide, superior alignment of DAMMIF models to HLA-E crystal structures was only achieved for RL9HIV-, Mtb14-, RL9SIV-, and IL9-HLA-E material when molar excess peptide was present throughout both HPLC injection and elution, implicating “weak” peptide-binding affinity as key contributing factors to the observed differences.

**Figure 2:**
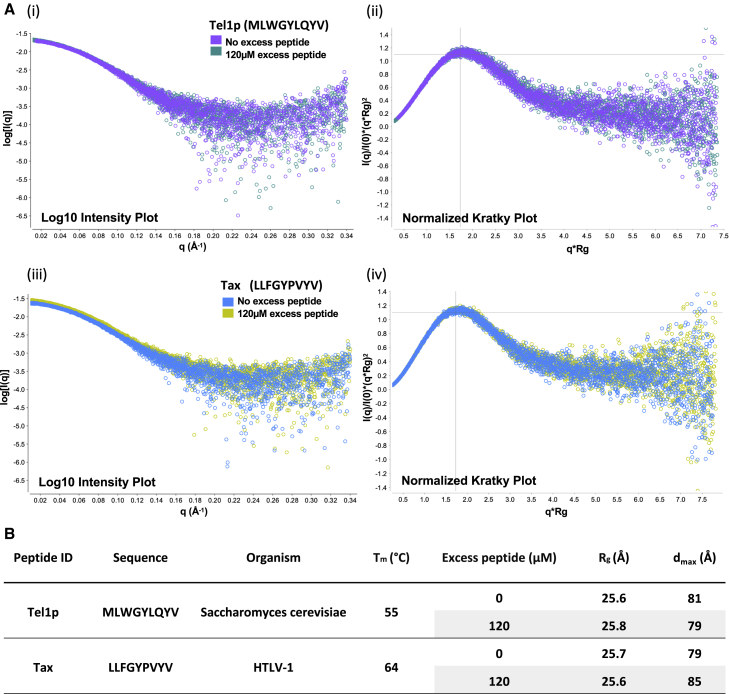
SEC-SAXS analysis for peptide-HLA-A2 complexes (A) (i) and (iii): Log_10_ scattering intensity plots for HLA-A^∗^02:01 SEC-SAXS experiments. The scattering vector q (Å^−1^) is plotted (x axis) versus scattered intensity, I(q), log scale (y axis). Scattering intensity curves for HLA-A2 refolds run with or without 120 μM excess peptide during elution, are plotted. (ii) and (iv): Normalized Kratky plots with superimposed curves corresponding to HLA-A2 SEC-SAXS experiments with or without 120 μM excess peptide. Modulated Gaussian curves are color-coded according to figure legends in log_10_ intensity plots. The scattering vector multiplied by the radius of gyration is plotted (x axis) versus the scattering intensity I(q) divided by the experiment’s I(0) and multiplied by (q^∗^R_g_)^2^ (y axis). (B) HLA-A^∗^02:01-peptide refolds tested via SEC-SAXS. Each row represents SEC-SAXS experiments conducted in the absence or presence of 120 μM excess peptide. The radius of gyration (R_g_) and maximum dimension (d_max_), both measured in Å, are specified, along with circular dichroism-measured T_m_ values (Borbulevych et al., 2009).

For comparison, SEC-SAXS experiments were performed for HLA-A^∗^02:01 refolded with the Tel1p (MLWGYLQYV) and Tax (LLFGYPVYV) epitopes derived from *S. cerevisiae* and HTLV-1, respectively (Figure 2) (Borbulevych et al., 2009; Hawse et al., 2013; Khan et al., 2000). Similar to VL9-refolded HLA-E, the shape of the scattering intensity curve and positioning of the modulated Gaussian peak on the normalized Kratky plot were unaffected by excess peptide addition (Figure 2A). Also, normalized Kratky plots revealed that HLA-A2 refolds obey the Guinier’s approximation, indicative of compact, fully folded globular protein. Notably, corresponding modulated Gaussian curves for VL9-refolded HLA-E both in the presence or absence of excess peptide exhibited slight deviations from this maximum, suggestive of a less compact species with a greater degree of conformational asymmetry or flexibility, relative to HLA-A2-peptide complexes (Figures 1B and 2A, ii and iv). Yet overall, HLA-E-bound VL9 is behaving like typical MHC Ia-peptide complexes, in contrast to the weaker binding pathogen-derived peptides.

### Correlations between SAXS data, HLA-E peptide-binding signals, and melting temperatures

**Figure 3:**
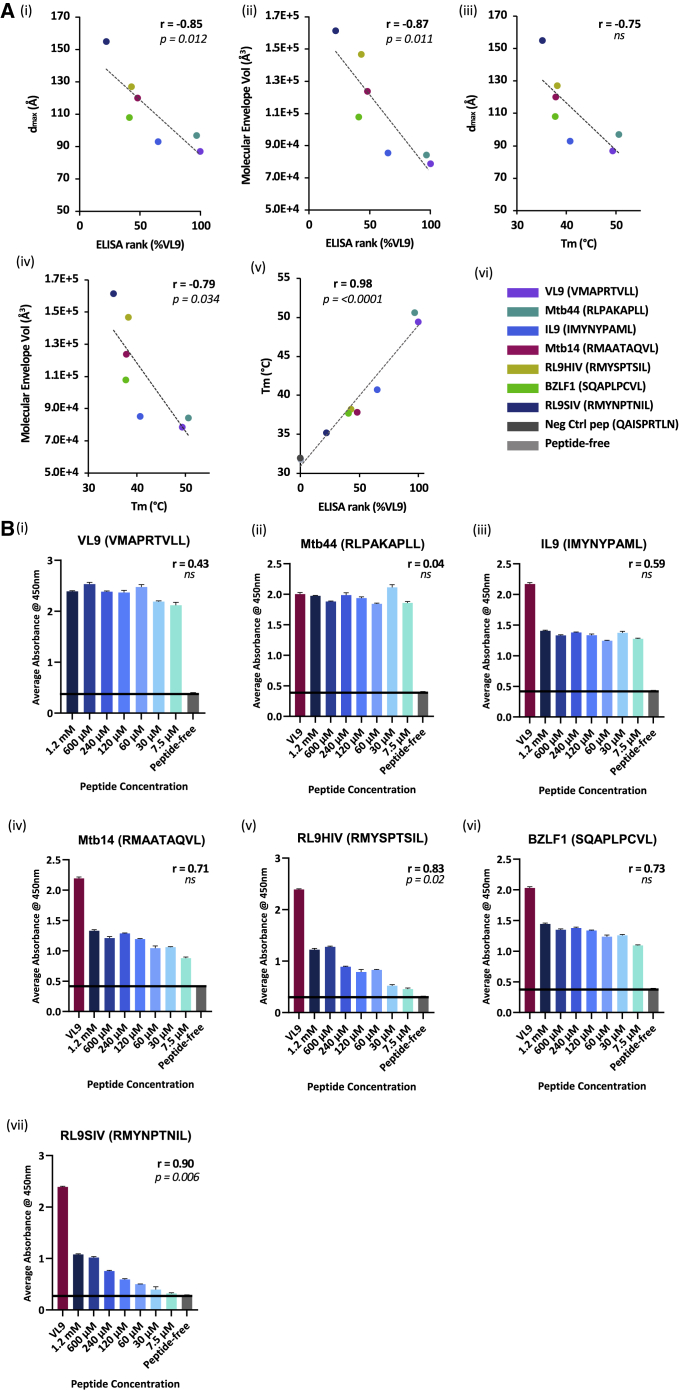
DAMMIF model structural alignment negatively correlates with HLA-E thermal stability and peptide-binding signals (A) (i–iv): Scatter graphs where ELISA-derived HLA-E peptide-binding signals or T_m_ values are plotted on the x axis versus SEC-SAXS-obtained d_max_ values or molecular envelope volumes (denoted in Å), on the y axes. (v): Scatter graph with normalized ELISA-based HLA-E peptide-binding signals and T_m_, denoted in A, plotted on the x and y axes, respectively. (vi): Figure legend for A (i–v). (B) (i–vii): Bar charts representing ELISA-based HLA-E peptide titration assays, with peptide concentration (x axis) versus average absorbance signals at 450 nm (y axis). The “peptide-free” negative control (dark gray) reflects the “no-rescue” exchange reaction. For (ii–vii), the positive control VL9 (VMAPRTVLL) peptide (dark red) corresponds to a 120 μM excess peptide exchange reaction. For (i–vii), Pearson’s correlation coefficients (r) are denoted, with corresponding p values or ns for non-significant correlations. Error bars depict SEM. Biological repeats, n = 3; technical replica n = 2.

Differential scanning fluorimetry (DSF)-generated melting temperatures (T_m_) were obtained for purified peptide-free-HLA-E complexes incubated with peptides from the intermediate binding panel in addition to a positive (VL9, VMAPRTVLL) and negative (HIV Gag, QAISPRTLN) control peptide at a 12 M excess peptide-to-protein ratio (Table S1). The negative control HIV Gag peptide generated a comparable T_m_ (31.8°C) to the peptide-free-HLA-E background (32.0°C), whereas Mtb44 generated a comparable T_m_ (50.6°C) to VL9-incubated HLA-E (49.4°C). The remaining peptides generated T_m_ values ranging from 35.2°C (RL9SIV) to 40.7°C (IL9). A strong positive correlation (r = 0.98) was observed between HLA-E complex T_m_ and previously obtained %VL9-binding scores from ELISA-based screens, highlighting the strong agreement between these independent techniques (Figure 3A). As immunogenic MHC Ia-restricted peptides more typically exhibit a higher range of T_m_ values, these HLA-E restricted intermediate binding epitopes measured low on the thermal-stability continuum.

Peptide titration assays were also conducted in the sandwich ELISA-based HLA-E peptide-binding assay (Figure 3B). Weak correlations were identified between peptide concentration and ELISA signals for the strongest binding peptides VL9 and Mtb44, indicating that these peptides support HLA-E complex formation at low concentrations (Figures 3B, i and ii). Stronger positive correlations were observed for the remaining intermediate pathogen-derived peptides, illustrating the weaker binding affinity of these epitopes (Figures 3B, iii–vii). Notably, ELISA signals for the VL9 and Mtb44 peptides remained significantly higher than those generated by the intermediate pathogen-derived peptides IL9, Mtb14, RL9HIV, BZLF1, and RL9SIV, even at higher concentrations (1.2 mM). Such observations not only demonstrate the exquisite selectivity of HLA-E for canonical VL9 but also reveal suboptimal HLA-E binding to these pathogen-derived epitopes.

Both T_m_ values and normalized ELISA signals exhibited strong negative correlations with SEC-SAXS-based measurements, including the maximal linear dimension (indicated by d_max_ values measured in Å) and DAMMIF model volume (measured in Å^3^) of HLA-E complexes in solution (Figures 3A, i–iv). Thus, as T_m_ and ELISA signals increase, the average conformation of the protein ensemble in solution contracts and more optimally aligns to its respective HLA-E crystallographic coordinates. By contrast, lower thermal stability of peptide-HLA-E complexes (T_m_ ≤ 40.7°C) correlates with increasingly poor global alignment of SAXS data to HLA-E crystal structures.

### Structural determination of mycobacterial epitope-bound HLA-E

**Figure 4:**
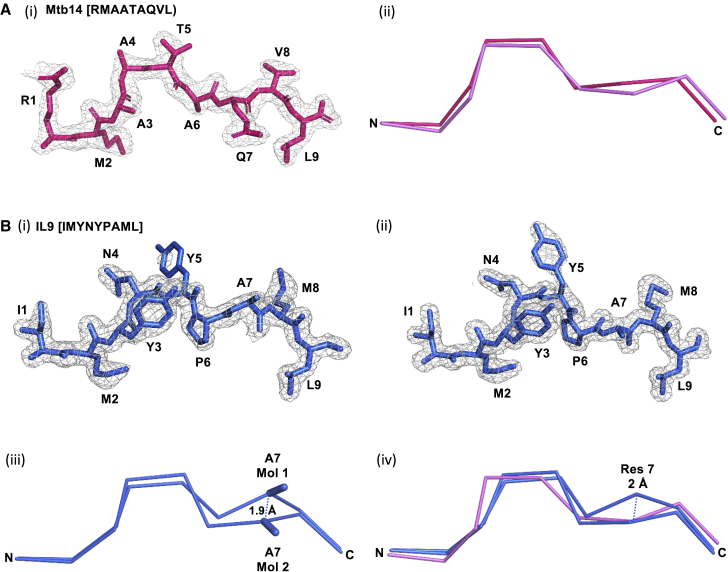
Structural characterization of mycobacterial peptide binding to HLA-E (A) (i): PyMol visualization (side-on) of peptide Mtb14, from molecule 1 of the asymmetric unit (hot pink stick form). An electron density map contoured to 1 sigma is displayed (gray mesh) overlaying the peptide. (ii): Ribbon representation of the peptide backbone (hot pink) from the HLA-E-Mtb14 structure with a superimposed VL9 peptide backbone (PDB: 1MHE, violet). The peptide N and C termini are labeled for clarity. (B) (i): Side-on visualization of peptide IL9, from molecule 1 of the asymmetric unit (blue stick form). (ii): Side-on visualization of peptide IL9 from molecule 2 of the asymmetric unit (blue stick form). In B (i) and (ii), an electron density map contoured to 1 sigma is displayed (gray mesh) overlaying the peptide. (iii): Superimposed peptide backbones are depicted with the N and C termini labeled. The IL9 peptide backbones from molecules 1 and 2 of the asymmetric unit (blue ribbon) with the position 7 A side chains (blue stick form) are reported. The distance (1.9 Å) separating the position 7 Cα atom of the IL9 peptides from molecules 1 and 2 of the asymmetric unit is denoted. (iv): Superimposed peptide backbones are depicted in ribbon form with the N and C termini labeled. The IL9 peptide backbones from molecules 1 and 2 of the asymmetric unit are displayed (blue), with the VL9 peptide backbone from the published HLA-E-VL9 structure (PDB: 1MHE) in violet. The 2.0 Å distance separating the IL9 position 7 Cα atom from molecule 1 of the asymmetric unit and the VL9 position 7 Cα atom is denoted.

The HLA-E^∗^01:03 heavy-chain extracellular domain and β2M were crystallized in complex with the HLA-E-restricted mycobacterial epitopes IL9 (IMYNYPAML) and Mtb14 (RMAATAQVL) (Joosten et al., 2010; McMurtrey et al., 2017). Data collection and refinement statistics are listed in Table S2. The HLA-E^IL9^ and HLA-E^Mtb14^ crystals diffracted X-rays to 1.7 Å and 2.05 Å, respectively. Four non-crystallographic symmetry-related molecules were present in each asymmetric unit, and packing occurred in the C2 space group. Clear, unambiguous electron density enabled manual model building of the mycobacterial epitopes into the HLA-E PBG (Figure 4). Mtb14 adopted the classical conformation akin to VL9, with a solvent-exposed-central kink at positions 4 and 5 (Figure 4A). The less constrained central kinked region of Mtb14 displayed the greatest movement relative to VL9 with 1.6 and 1.7 Å separating Cα atoms at positions 4 and 5, respectively, resulting in the central portion of the Mtb14 backbone projecting closer toward the HLA-E α1 helix.

Unusually, conformational peptide dimorphism was observed for IL9, which adopted two distinct configurations in different non-crystallographic symmetry (NCS)-related molecules of the asymmetric unit (Figure 4B). One such configuration, in two NCS-related molecules, closely aligned to VL9 peptides. However, an alternative conformation in the two remaining NCS-related molecules, featuring adjustments of the peptide backbone at position 7, resulted in divergent trajectories of the A side chains (Figures 4B, iii and iv). In the alternative IL9 peptide configuration, the Cα atom at position 7 projected 2 Å further toward the solvent relative to VL9. This arrangement led to the secondary anchor side chain swinging away from its corresponding E pocket.

### Comparative structural analyses reveal a crucial role for the E pocket in complex stability

**Figure 5:**
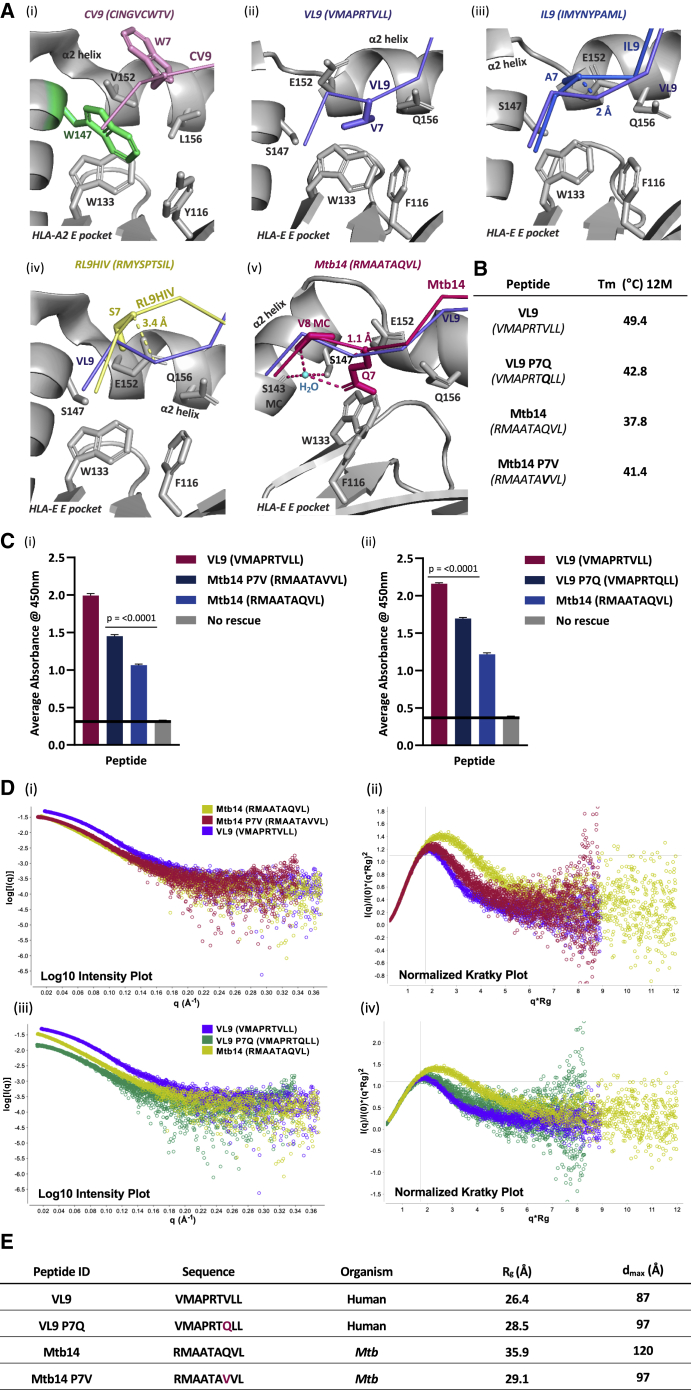
Suboptimal E pocket interactions compromise HLA-E complex stability (A) (i): HLA-A2 E pocket (PDB: 3MRG [Reiser et al., 2014]) visualization with side chains of pocket-forming residues depicted (gray or green sticks). The hepatitis C virus-derived CINGVCWTV peptide (pink ribbon) with the solvent-exposed position 7 side chain (stick form) projecting away from the E pocket. HLA-A2 groove regions including the α2 helix and β-sheet floor are shown (gray cartoon). (ii–v): Position 7 anchor side-chain-accommodating E pocket of HLA-E. E-pocket-forming residue side chains are depicted (gray sticks) with remaining regions of the PBG (gray cartoon). In (ii), the VL9 peptide main chain (purple ribbon) with the position 7 side chain (purple stick-form) projecting downward into the secondary E pocket is shown (PDB: 1MHE). (iii–v): Illustration of differential positioning at position 7 of HLA-E-bound pathogen-derived peptides relative to VL9 (PDB: 1MHE). The IL9, RL9HIV, and Mtb14 peptide backbones are depicted (blue [iii], yellow [iv], and magenta [v] ribbons, respectively), with position 7 side chains: Ala-7 of IL9, Ser-7 of RL9HIV, and Gln-7 of Mtb14 (blue [iii], yellow [iv], and magenta [v] stick form, respectively). The superimposed VL9 peptide main chain (PDB: 1MHE) is shown (purple ribbon), and the distance between the aligned peptide position 7 Cα atoms is indicated by dashed lines for the following: 2 Å for VL9 and IL9, 3.4 Å for VL9 and RL9HIV, and 1.1 Å for VL9 and Mtb14. In (v), water-mediated inter-chain hydrogen bonds (magenta dashed lines) with the coordinated H_2_O molecule (cyan-shaded sphere) are depicted. Four hydrogen bonds, which indirectly link the Mtb14 position 7 Gln side chain to the HLA-E α2-helix Ser-147 side chain and Ser-143 main chain, in addition to the Mtb14 peptide position 8 main chain, are shown. The Mtb14 Val-8 main chain and HLA-E Ser-143 main chain are labeled “MC” (magenta and gray sticks, respectively). (B) HLA-E-β2M-peptide thermal stability (T_m_) in the presence of 12 M excess peptide. T_m_ values (°C) are listed for VL9, Mtb14, and the corresponding position (p)7 variant peptides VL9 p7Q and Mtb14 p7V. (C) (i): Bar chart of an ELISA-based HLA-E peptide-binding assay in which the wild-type Mtb14 peptide was compared with the Mtb14 p7V peptide. The VL9 peptide-positive control (dark red) and the negative peptide-free “no rescue” control (gray) are indicated. Test peptides are plotted (x axis) versus the average absorbance readings at 450 nm (y axis); p values from unpaired t tests are denoted no asterisk p > 0.05, ^∗^p ≤ 0.05, ^∗∗^p ≤ 0.01, ^∗∗∗^p ≤ 0.001, ^∗∗∗∗^p ≤ 0.0001. Error bars depict SEM. Biological repeats, n = 3; technical replica, n = 2. (ii): Bar chart as described in (C) (i), in which HLA-E binding to VL9 was compared with a variant peptide containing p7Q. The Mtb14 peptide, which naturally contains p7Q, was included for reference. (D) (i) and (iii): Log_10_ scattering intensity plots for HLA-E SEC-SAXS experiments. Plotted on the x axis is the scattering vector q (Å^−1^) with scattered intensity, I(q), log scale on the y axis. Superimposed scattering intensity curves for peptide-HLA-E refolds are color coded according to the corresponding figure legend. (ii) and (iv): Normalized Kratky plots with superimposed curves from HLA-E SEC-SAXS experiments. Superimposed modulated Gaussian curves are color coded according to legends in log_10_ intensity plots. The scattering vector multiplied by the radius of gyration (x axis) is plotted versus the scattering intensity I(q) divided by the experiment’s I(0) and multiplied by (q^∗^R_g_)^2^ (y axis). (E) Summary detailing HLA-E-peptide refolds tested via SEC-SAXS. Peptide ID, sequence and origin are specified along with the radius of gyration (R_g_) and maximum dimension (d_max_), both measured in Å.

A comparative structural analysis of HLA-E revealed that disrupted E pocket occupancy featured exclusively among structures whose corresponding SEC-SAXS-obtained DAMMIF models appeared elongated, including HLA-E^IL9^, HLA-E^Mtb14^, and the published HLA-E^RL9HIV^ structure (Walters et al., 2018). Canonical primary anchor residues, position 2 M and position 9 L, are present in IL9 (IMYNYPAML), Mtb14 (RMAATAQVL) and RL9HIV (RMYSPTSIL) and projected into their respective B and F pockets similarly to that observed for the more conformationally stable HLA-E^VL9[VMAPRTVLL]^ complex (Hoare et al., 2008; O’Callaghan et al., 1998; Strong et al., 2003) Therefore, unmet peptide-binding criteria featuring suboptimal E pocket-based interactions most likely contributed to the conformational heterogeneity and complex instability detected by SEC-SAXS for HLA-E^IL9^, HLA-E^Mtb14^, and HLA-E^RL9HIV^. The HLA-E secondary E pocket comprises a deep, hydrophobic, pocket-like recess in contrast to MHC Ia molecules, which have a large, highly conserved position 147 W side chain that occludes this region (Figures 5A, i). The larger E pocket of HLA-E accommodates downward-projecting, non-polar peptide residues at position 7, V or L, which are conserved among VL9 variants (Figures 5A, ii). The unusual conformational dimorphism of the IL9 peptide primarily involves repositioning of the position 7 A Cα atom (by 2 Å) further toward solvent relative to VL9 in one of the two observed peptide configurations (Figures 4B) (Figure 5A, iii). Given that HLA-E-bound peptides including IL9 commonly participate in crystal-packing interfaces, such structural polymorphism may reflect weak tethering of the position 7 A side chain to the deep secondary E pocket, rendering it susceptible to crystal packing-induced repositioning. Similarly, an unoccupied E pocket is a distinct feature of the previously published HLA-E^RL9HIV^ complex structure, with the position 7 S Cα atom projecting 3.4 Å further toward solvent relative to HLA-E^VL9[VMAPRTVLL]^ (Figures 5A, iv) (Walters et al., 2018). The resulting disruptions to stabilizing E pocket-based interactions with potentially destabilizing E pocket-based cavities thus ensue for HLA-E^IL9^ and HLA-E^RL9HIV^ complexes (Xu et al., 1998). A related phenomenon was also observed for Mtb14-bound HLA-E. The polar Q at position 7 of Mtb14 is buried within the hydrophobic E pocket and forms water-mediated hydrogen bonds to HLA-E α2 helix residues S143 and S147 (Figure 5A, v). Combined peptide mutagenesis and ELISA-based HLA-E peptide binding or thermal-melt assays support a major contribution of optimal E pocket occupancy by the peptide’s position 7 side chain to complex stability; the introduction of a canonical non-polar residue (V) at position 7 in place of a polar side chain (Q) in Mtb14 (Mtb14 P7V) resulted in a 36% increase in the ELISA-based binding signal and a 3.6°C increase in thermal stability (Figures 5B and 5C). By contrast, the introduction of a polar Q at position 7 of VL9 (VL9 P7Q) yielded a 23% reduction in the ELISA-based binding signal, with an additional 6.6°C drop in thermal stability. Analogously, SEC-SAXS analysis of the HLA-E^Mtb14 P7V^ complex resulted in a 6.8 Å decrease in the R_g_, a 23-Å decrease in the d_max_, and a shifted modulated Gaussian curve peak on the normalized Kratky plot, which was positioned closer to q^∗^Rg = √3 with a magnitude of 3·e−1 relative to wild-type Mtb14-bound HLA-E, indicative of a more globular and compact average conformer (Figures 5D and 5E). By contrast, SEC-SAXS analyses of HLA-E^VL9 P7Q^ resulted in an increased R_g_ and d_max_ in addition to a shifted curve on the normalized Kratky plot, suggesting a greater degree of conformational flexibility or asymmetry relative to wild-type VL9-bound HLA-E, and further validating the importance of E pocket occupancy for optimal complex stability.

### Distinct structural features distinguish VL9 versus pathogen peptide-bound HLA-E

**Figure 6:**
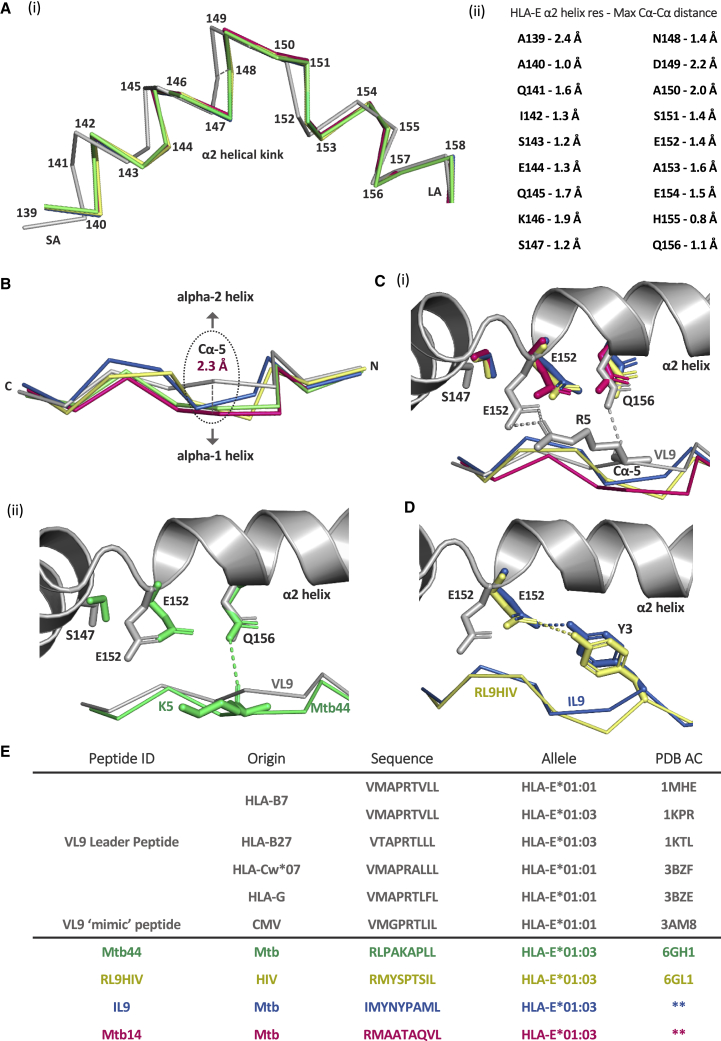
Distinct structural motifs emerge in the absence of HLA-E-bound VL9 peptide (A) (i): Superimposed HLA-E α2 helical kink regions depicted as lines with the α2 helix short-arm labeled “SA,” the long-arm labeled “LA,” and residue positions denoted. The HLA-E-VL9 α2 helix (PDB: 1MHE) is shaded gray. Superimposed pathogen peptide-bound HLA-E α2 helices are depicted, including HLA-E-Mtb44 (green), HLA-E-RL9HIV (yellow), HLA-E-Mtb14 (magenta), and HLA-E-IL9 (blue). (ii): The maximum distance in Å separating Cα atoms of HLA-E-VL9 α2 helix residues (1MHE) versus corresponding Cα atoms in pathogen peptide-bound HLA-E structures. (B) Aligned HLA-E-bound peptide Cα backbones are depicted as ribbons following whole HLA-E complex superposition. The VL9 peptide (PDB: 1MHE) is shaded gray. Pathogen-derived HLA-E-bound peptides are colored green (Mtb44), yellow (RL9HIV), magenta (Mtb14), and blue (IL9). HLA-E α1 and α2 helices plus N and C peptide termini positions are indicated. Position 5 Cα atoms are circled, and the maximum distance separating the VL9 peptide Arg-5 Cα from pathogen peptide position 5 Cα atoms are denoted and measure 2.3 Å for VL9 and RL9HIV, 1.9 Å for VL9 and IL9, 1.9 Å for V9 and Mtb14, and 1.5 Å for VL9 and Mtb44. (C) (i): Visualization of the HLA-E α2 helix (gray cartoon) with aligned pathogen-derived peptide backbones RL9HIV, IL9, and Mtb14, in yellow, blue, and magenta ribbon, respectively. The superimposed VL9 peptide backbone (PDB: 1MHE) is shown in gray ribbon. The two salt bridges connecting HLA-E Glu-152 and the Arg-5 side chain of the VL9 peptide are shown (gray dashed lines). The HLA-E Gln-156 side chain-VL9 Arg-5 oxygen main-chain hydrogen bond is also depicted (gray dashed lines). (ii): The HLA-E α2 helix is shown (gray cartoon) with the aligned Mtb44 (green) and VL9 (PBD: 1MHE[gray]) peptide backbones. Molecule 3 of the HLA-E-Mtb44 structure, in which a hydrogen bond (green dashed line) connects the HLA-E Gln-156 side chain to the main chain oxygen of Mtb44 Lys-5 (green sticks) is depicted. This hydrogen bond is present in one of the four molecules in the asymmetric unit. For C (i) and (ii), HLA-E α2 helix Ser-147, Glu-152, and Gln-156 side chains (stick form) are color-coded according to the corresponding peptide. (D) The HLA-E α2 helix is shown (gray cartoon) with the aligned RL9HIV and IL9 peptides (yellow and blue ribbon, respectively). The RL9HIV and IL9 Tyr-3 side chains are shown in yellow and blue stick form, respectively. HLA-E α2 helix Glu-152 side chains from the HLA-E-RL9HIV and HLA-E-IL9 structures are shown (yellow and blue sticks, respectively) with corresponding hydrogen bonds (yellow/blue dashed lines). The HLA-E-VL9 Glu-152 side chain (PBD : 1MHE) is shown in gray stick form for reference. (E) Details of published HLA-E-peptide structures plus the 2 HLA-E structures presented here. Peptide IDs, origins, and amino acid sequences are specified with the corresponding HLA-E allotype and PDB accession codes.

Comparative structural analyses revealed a distinct configuration located in the α2-helical kink region, which distinguishes VL9-versus pathogen peptide-bound HLA-E complexes (Figure 6A) (Figure S4) ). Multiple polar interactions between the HLA-E α2 helix and the central regions of the VL9 peptide contribute to HLA-E-VL9 conformational stability. The Q156 of the HLA-E α2 helix forms a hydrogen bond with the position 5 main-chain carbonyl of VL9. In addition, the VL9 R5 side chain mediates multiple ionic contacts with the HLA-E α2 helix E152 residue (Figure S4A, ii and iii). In contrast, structural rearrangement of the position 5 peptide main chain of up to 2.3 Å toward the α1 helix, relative to VL9, was observed for all pathogen peptides (Figure 6B). This, in addition to the absence of a position 5 R side chain, resulted in the loss of these centrally focused, intermolecular interactions (Figure 6C), thereby facilitating disruption in the local α2-helical geometry and side-chain configurations. Positional adjustment of the HLA-E α2-helical mainchain (up to 2.4 Å) and side chains (up to 4 Å) between residues A139 and Q156 marks structures of HLA-E in complex with pathogen epitopes relative to VL9-bound HLA-E (Figures 6A–6C) (Figure S4A, v–xi). Interestingly, in one of four molecules of the asymmetric unit for HLA-E^Mtb44^, the hydrogen bond connecting Q156 to the main chain carbonyl of position 5 of the Mtb44 peptide was preserved (Figures 6C, ii). As a result, Q156 positioning in this HLA-E-Mtb44 molecule was not disrupted and aligned well with the corresponding region of HLA-E-VL9 structures. As Mtb44 was the only pathogen peptide that exhibited comparable binding to VL9 *in vitro,* this centrally positioned hydrogen-bonding interaction, albeit partially preserved, could play a critical role in HLA-E complex stability (Walters et al., 2020). In the remaining pathogen epitope-bound HLA-E structures, the loss of this hydrogen bond resulted in the Q156 side chain undergoing conformational rearrangement of up to 2.2 Å (Figures 6C, i). Additionally, in all pathogen peptide-HLA-E structures, both the backbone and the side chain of S147 adopted distinct configurations of up to 1.9 Å relative to VL9-bound HLA-E (Figure 6C). However, most strikingly, the absence of the peptide R5-HLA-E E152 salt bridge allowed E152 to reorient up to 4 Å in all pathogen peptide-HLA-E structures, including the HLA-E^Mtb44^ complex (Figures 6C and S4). This rearrangement drove the emergence of an alternative peptide-binding motif for HLA-E molecules in complex with non-VL9 peptides bearing Y at position 3. More specifically, the E152 side chain in HLA-E^IL9^ and HLA-E^RL9HIV^ complexes protruded toward the N terminus of the peptide-binding groove and mediated inter-chain hydrogen bonding with the hydroxyl group of position 3 Y (Figure 6D). As VL9 peptides contain a highly conserved position 3 A residue, which projects into the shallow secondary D pocket, these data redefine amino acids that can be stably accommodated at the secondary anchor position 3 of non-VL9 HLA-E-binding peptides. It also provides an example of compensatory peptide-HLA-E intermolecular interactions which circumvent sub-optimal fitting of secondary anchor residues in the D pocket. Notably, all three differentially positioned residues, S147, E152, and Q156, are conserved among human, murine, and rhesus MHC-E molecules (Table S3).

α3 domain structural polymorphisms constitute another shared feature among pathogen-derived epitope-associated HLA-E complexes; intra-structural α3 domain rigid-body shifts are observed with up to 2.9Å separating corresponding Cα atoms of α3 domain β-strands in NCS-related molecules within the asymmetric units of HLA-E^Mtb44^, HLA-E^IL9^, HLA-E^RL9HIV^, and HLA-E^Mtb14^ (Figure S5) (Walters et al., 2018). Such structural polymorphisms, also reported occasionally for certain MHC Ia structures (Borbulevych et al., 2009; Riley et al., 2018; Smith et al., 1996) likely reflect reduced positional coordination and increased flexibility of the α3 domain in solution, which may in turn render this subunit susceptible to crystal-packing-induced repositioning. By contrast, superposition of NCS-related molecules from previously published structures of HLA-E-bound VL9 variants revealed strong alignment in α3 domain positioning (Figure S5) (Hoare et al., 2008; O’Callaghan et al., 1998; Petrie et al., 2008; Strong et al., 2003; Sullivan et al., 2007).

## Discussion

In light of recent findings reporting the unprecedented success of vaccine-induced MHC-E-restricted CD8^+^ T cells conferring sterile immunity against SIV challenge in rhesus macaques (Malouli et al., 2021), we conducted a structural and biophysical characterization for HLA-E in complex with pathogen-derived epitopes versus VL9. These pathogen-derived epitopes comprised the strongest non-VL9 HLA-E-binding peptides previously identified in ELISA-based screens (Walters et al., 2020). SEC-SAXS analyses revealed marked differences in the HLA-E conformational ensemble between VL9 versus pathogen-derived peptide bound forms. The latter yielded conformationally heterogeneous populations and DAMMIF models with poor alignment to HLA-E crystal structures. Such conformational heterogeneity and decreased globularity likely indicate a greater degree of complex instability and is reminiscent of conformationally flexible, partially peptide-loaded MHC Ia transition states (Bailey et al., 2015; Yanaka et al., 2014). Accordingly, there was a strong negative correlation between the extent of DAMMIF model versus structure misalignment and HLA-E complex thermal stability or *in vitro* peptide binding. By contrast, HLA-E-VL9 refolds yielded conformationally homogeneous protein ensembles with compact DAMMIF models that superposed to HLA-E crystallographic coordinates. Notably, the capacity of excess peptide to transform poorly aligned DAMMIF models into strongly aligned compact forms resembling VL9-bound HLA-E, implicates weak peptide binding as the key contributor to misalignment. These constitute unusual features for highly immunogenic epitopes such as the immunodominant RL9SIV (RMYNPTNIL) vaccine-identified “supertope,” which elicited Mamu-E-restricted CD8^+^ T cell responses in all rhesus macaques immunized with the RhCMV68-1-vectored SIV vaccine. Surprisingly, the majority of immunogenic MHC-E-restricted SIV epitopes gave very weak binding signals in ELISA screens (<10%VL9), including the second immunodominant SIV supertope, EK9 (EKQRESREK). Similarly, the mycobacterial epitope EK11 (EIEVDDDLIQK), which elicits stronger HLA-E-restricted CD8^+^ T cell responses than the IL9 epitope (McMurtrey et al., 2017), produced minimal binding in the ELISA assay. Unfortunately, small HPLC protein elution peaks generated by HLA-E refolded with these peptides induced insufficient X-ray scattering for reliable downstream SEC-SAXS data processing. In the absence of molar excess peptide, the intermediate binding HLA-E epitopes identified from ELISA-based screens provide insufficient binding energy to drive homogeneous fully folded protein complexes, whereas the weak binding immunogenic epitopes drive insufficient protein refolding for SAXS-based analysis. Nevertheless, the finding that these peptides can be immunodominant MHC-E-restricted epitopes implies that some form of antigen presentation *in vivo* is sufficient to prime T cells. This suggests that immunogenicity of MHC-E-restricted epitopes does not easily equate to optimal complex formation *in vitro*, a finding at odds with the positive correlation that generally relates immunogenicity to complex stability for MHC Ia molecules (Harndahl et al., 2012). This raises the question of whether MHC-E-restricted CD8^+^ T cells might be mechanistically distinct from their classically restricted counterparts and recognize alternative structural forms that arise in the absence of VL9.

Structural determination of HLA-E in complex with the intermediate binding IL9 and Mtb14 peptides, in addition to the previously published HLA-E^RL9HIV^ structure, aligned well with VL9-bound HLA-E structures and contrasted with the poorly aligned corresponding DAMMIF models (Walters et al., 2018). Crystallographic analyses failed to detect such conformationally flexible in-solution forms due to preferential incorporation of the lowest-energy state into the crystal, with subsequent lattice-imposed restraints. Thus, crystal structures of IL9-, Mtb14-, and RL9HIV-bound HLA-E likely represent a subpopulation of the heterogeneous ensembles observed via SEC-SAXS. Despite indistinguishable global architectures for pathogen peptide-HLA-E structures following HLA-E-VL9 superposition, differential local structural features were identified and may be linked to complex instability, as indicated by the poorly aligned DAMMIF models. IL9, Mtb14, and RL9HIV peptides include canonical primary anchor residues at position 2 (M) and position 9 (L), which optimally occupy the B and F pockets, respectively. However, in contrast to VL9, disrupted occupancy of the larger, secondary E pocket is a characteristic feature of all three pathogen peptide-HLA-E structures. A key stabilizing role for the E pocket was confirmed, as HLA-E refolded with an Mtb14 variant incorporating a position 7 Q-to-V substitution resulted in increased thermal stability and a retracted DAMMIF model. The requirement for optimal E pocket occupancy in addition to the primary B and F pockets for HLA-E aligns with its specific composition relative to MHC Ia; W147 is highly conserved among MHC Ia molecules, with its bulky side chain largely occluding the E pocket. In nearly all MHC Ia, a hydrogen bond is formed between W147 and the carbonyl group of the peptide’s final peptide bond (Elliott et al., 1993). This orients the penultimate side chain upward toward the TCR while also stabilizing peptide binding. In contrast, the smaller S147 present in HLA-E, and in its murine (Qa-1) and rhesus (Mamu-E) counterparts, results in a deep hydrophobic recess with a discrete pocket (O’Callaghan et al., 1998). The more pronounced E pocket likely serves to impose additional peptide-binding criteria that must be satisfied both to obtain optimal complex stability and to limit HLA-E peptide diversity by promoting a VL9-dominated ligandome to maintain CD94/NKG2A-mediated sensing by NK cells.

The lack of optimal E pocket occupancy—displayed by the majority pathogen-derived peptides described here—most likely destabilized the adjacent α2 region and contributed to the differentially positioned α2-helical kink that discriminated pathogen peptide-from-VL9-bound HLA-E. This configuration, primarily involving E152, could conceivably facilitate immune discrimination between VL9- versus non-VL9-bound HLA-E in healthy and pathological states, respectively. E152 is a common TCR-interacting residue, and its substitution to A results in a >10-fold reduction in CD94/NKG2A binding (Sullivan et al., 2007). Hence, exclusive E152 repositioning in structures of HLA-E lacking VL9 could signal this loss to both HLA-E-restricted CD8^+^ T cells and NK cells in an innate-like manner, not wholly dictated by peptide-specific interactions (Kaiser et al., 2008; Petrie et al., 2008; Sullivan et al., 2007). Accordingly, semi-invariant Qa-1-restricted T cells with a common Vα recognized non-Qdm (MHC Ia signal) TAP-independent peptides presented by Qa-1 (Doorduijn et al., 2018). Innate-like T cell recognition has previously been reported for other unconventionally restricted subsets including CD1d-restricted semi-invariant natural killer T (iNKT) cells and MR1-restricted mucosal-associated invariant T (MAIT) cells (Cotton et al., 2018). A few studies have demonstrated distinct modes of CD1-restricted TCR recognition that transcend the epitope/MHC co-recognition paradigm, with minimal direct contact between TCR and CD1-associated lipid antigen (Birkinshaw et al., 2015; Wun et al., 2018). Whether specific TCR α or β chains employ invariant regions to recognize HLA-E-bound pathogen-derived peptides is unknown. The distinct E152 orientation shared among HLA-E structures in complex with pathogen peptide described here also evolves the permitted HLA-E peptide-binding motif. Although VL9 peptides contain a highly conserved position 3 A that projects into the shallow D pocket, E152 repositioning plus the hydrogen bond formation with the position 3 Y side chains of HLA-E-bound IL9 and RL9HIV redefines what can be accommodated at the position 3 anchor. Such distinct intermolecular hydrogen bonding appears to compensate for a lack of peptide side-chain shape complementarity with its corresponding D pocket. An identical out-of-pocket peptide side-chain-binding mechanism with conserved intermolecular hydrogen bonding was previously reported for H2-Kb and a position 3 Y-containing octameric vesicular stomatitis virus-derived peptide (Fremont et al., 1992), with an H2-Kb E152-to-A mutation abrogating peptide binding (van Bleek and Nathenson, 1991). Conservation of E152 in H2-Kb and all known human, rhesus, and murine MHC-E alleles is unusual, with A or V predominantly featuring at position 152 in MHC Ia molecules (Table S3). Thus, although E152 is important in maintaining a salt bridge with R at position 5 of VL9, repositioning of E152 in pathogen peptide-bound HLA-E structures offers an alternative peptide-binding mode primarily involving Y at position 3 of the peptides, as shown here.

In summary, our results demonstrate that the stringent peptide-binding criteria imposed by HLA-E are optimally satisfied by the VL9 peptide. These restrictive binding requirements are likely driven by NK-cell evolutionary forces to maintain a VL9-dominated peptide repertoire, which in healthy cells maintains inhibitory NK cell interactions through CD94/NKG2A engagement. Non-VL9 HLA-E-restricted peptides with comparable or higher binding affinity could disrupt such immunoregulatory interactions. Consistent with this, our data suggest that diverse peptide sampling by HLA-E, most likely occurring when MHC Ia trafficking pathways that normally deliver VL9 are disrupted, is largely suboptimal. Such peptides yield less stable HLA-E complexes and produce α2-helical configurations that likely impact TCR and CD94/NKG2 receptor recognition.

### Limitations of the study

Collectively, our data offer detailed insights into key structural facets that distinguish HLA-E-VL9 and non-VL9 peptide binding, which generates an antigenic surface that discriminates pathogen peptide-bound HLA-E from HLA-E-VL9. We propose that this potentially leads to an immune recognition footprint more generally shared among pathogen-bound epitopes. However, we do not present either structural or biophysical data pertaining to the recognition of this footprint from a TCR perspective.

## STAR★Methods

### Key resources table

**Table:** 

REAGENT or RESOURCE	SOURCE	IDENTIFIER
**Antibodies**		

3D12	Biolegend	Cat#242602; RRID: AB_1659247
Rabbit anti-human b2m HRP	Thermo Scientific (Pierce)	Cat#PA1-29662; RRID: AB_1956329
Envision + System (goat anti-rabbit HRP)	Agilent	Cat#K4003; RRID: AB_2630375

**Bacterial and virus strains**		

BL21DE3pLysS	Merck	Cat#69451-3

**Chemicals, peptides, and recombinant proteins**		

Synthetic peptides	Genscript	https://www.genscript.com/
UV-labile peptide	The Leiden University Medical Center, Leiden, The Netherlands	https://ccb.lumc.nl/
ELISA Coating buffer(x5)	Biolegend	Cat#421701
ELISA Wash Buffer (x20)	Biolegend	Cat#421501
TMB High sensitivity Substrate	Biolegend	Cat#421501
Stop Solution	Biolegend	Cat#423001

**Critical commercial assays**		

NativePAGE™ Sample Buffer (4X)	ThermoFisher Scientific	Cat#BN2003
NativeMark™ Unstained Protein Standard	ThermoFisher Scientific	Cat#LC0725
NativePAGE™ 4 to 16%, Bis-Tris, 1.0 mm, Mini Protein Gel	ThermoFisher Scientific	Cat#BN2001
NativePAGE™ Cathode Buffer Additive (20X)	ThermoFisher Scientific	Cat#BN2002

**Deposited data**		

HLA-E^∗^01:03^IL9^ structural coordinates	https://www.rcsb.org	7P4B
HLA-E^∗^01:03^Mtb14^structural coordinates	https://www.rcsb.org	7P49

**Recombinant DNA**		

pGMT7-HLA-E^∗^01:03	This paper	N/A
pGMT7-β2m	This paper	N/A

**Software and algorithms**		

CCP4 Program Suite v7.1.018	CCP4 Research Complex at Harwell (RCaH)	https://www.ccp4.ac.uk/download/#os=wsl
PyMOL Molecular Graphics System, version 2.0	Schrödinger, LLC	https://pymol.org/2/
PHENIX	Lawrence Berkeley National Laboratory	https://phenix-online.org/
FlowJo version 10.7.1	Becton, Dickinson and Company Ashland. FlowJo was acquired by BD in 2017	https://www.flowjo.com/solutions/flowjo/downloads
FLUOstar OMEGA	BMG Labtech	https://www.bmglabtech.com/
PR.ThermControl, version 2.1.5	NanoTemper Technologies GmbH Flößergasse 4 81,369 München Germany	https://nanotempertech.com/
ATSAS 3.0.4	(Manalastas-Cantos et al., 2021)	https://www.embl-hamburg.de/biosaxs/download.html
MolProbity	Duke University	http://molprobity.biochem.duke.edu/
GraphPad Prism 7	GraphPad by Dotmatics	https://www.graphpad.com

### Resource availability

#### Lead contact

Further information and requests for resources and reagents should be directed to and will be fulfilled by the lead contact, Geraldine Gillespie (geraldine.gillespie@ndm.ox.ac.uk).

#### Materials availability

This study did not generate new unique reagents.

### Experimental model and subject details

Protein expression was performed using the commercially-available *E.coli* derivative, BL21(DE3)pLysS bacterial strain (genotype F^–^*omp*T *hsd*S_B_ (r_B_^–^, m_B_^–^) *gal dcm* (DE3) pLysS(Cam^R^). Handing of BL21(DE3)pLysS cells was performed in BSL2 approved facilities at the NDMRB, University of Oxford using appropriate personal protective equipment and following local risk assessed Standard Operation Protocols for the handling and disposal of genetically modified organisms.

### Method details

#### Peptide synthesis

Peptides purchased as lyophilised powder at >85% purity from Genscript USA were reconstituted in DMSO (200 mM) and stored at −80°C. A UV-labile peptide based on the HLA-B leader peptide (VMAPRTLVL) with a 3-amino-3-(2-nitrophenyl)-propionic acid residue (J residue) substitution at position 5 was obtained from LUMC, The Netherlands, for use in peptide binding assays.

#### Protein refolding

HLA-E^∗^01:03 or HLA-A^∗^02:01 proteins refolds were assembled in the traditional macro-refolding buffer for MHC class I molecules comprising 100 mM Tris pH8.0, 400 mM L-arginine monohydrochloride, 2 mM EDTA, 5 mM reduced glutathione and 0.5 mM oxidised Glutathione, prepared in MiliQ water. β2-Microglobulin in Urea-Mes, was initially refolded for 30 min at 4°C at a final concentration of 2 μM. Test peptide was subsequently added to the refold at a concentration of 30–120 μM followed by HLA-E^∗^01:03 or HLA-A^∗^02:01 heavy chain which was pulsed into the refolding buffer to reach a final concentration of 1 μM. Refolds were subject to a 72 h incubation period at 4°C prior to filtration through 1.0 μM cellular nitrate membranes to ensure the removal of aggregated material. Refolds were concentrated by a VivaFlow 50R system and VivaSpin Turbo Ultrafiltration centrifugal devices, both with 10 kDa molecular weight cut-offs. Refolded and concentrated material, with peptide concentrations of 120 μM, was used at a concentration of 10 mg/mL in SEC-SAXS experiments without subsequent chromatographic separation.

Refolded and concentrated material intended for ELISA-based HLA-E peptide binding assays, DSF or crystallisation screening was subject to subsequent fast protein liquid chromatography (FPLC) size separation on an AKTA Start System using a Superdex S75 16/60 column. HLA-E protein complex peaks were eluted into 20 mM Tris pH8, 100 mM NaCl and discriminated from non-associated β2M and large misfolded aggregates via elution profile visualisation by UV absorbance at 280 mAU. FPLC-purified protein peaks fractions were combined and concentrated to a desired concentration for subsequent experiments using 10 kDa cut-off VivaSpin Turbo Ultrafiltration centrifugal devices - the final protein concentration was obtained by measurement of the absorbance at 280 nm using a NanoDrop ND-1000 Spectrophotometer. The composition of eluted protein samples was also analyzed by non-reducing SDS-PAGE electrophoresis on NuPAGE 12% Bis-Tris protein gels to demonstrate the presence of non-aggregated HLA-E heavy chain and β2M.

#### Blue native gels

HLA-E-β2m complexes previously refolded with the UV-sensitive VL9 peptide (VMAPJTVLL) were incubated in the presence of molar excess test peptide and evaluated via the Blue Native-PAGE Novex Bis–Tris gel system (life technologies) (Walters et al., 2018). In brief, pre-refolded and purified HLA-E in complex with the UV-sensitive peptide was incubated for 3 h on ice in the presence of 12 M excess test peptide prior to the addition of 3 μL 4× Native-PAGE Sample Buffer per 10 μg (10 μL) of sample. Samples were loaded onto 4–16% Native-PAGE Novex Bis–Tris gels with NativeMark Unstained Protein Standard used as the ladder control. Gel electrophoresis was carried out at 150 Volts for 2 h at RT with a current gradient ranging from 15 to 16 to 2–4 mAmps. Gels were subsequently rinsed three times in MilliQ water and stained for 2–3 h in SimplyBlue SafeStain at RT. The MiliQ water was changed a number of times over a 24–48 h period to enable gel de-staining.

#### Size exclusion chromatography-coupled small angle X-ray scattering

125 mL HLA-E-β2M-peptide refolds were assembled in the L-Arginine-Tris macro-refolding buffer according to the protein refolding method detailed above and incubated for 72hrs at 4°C prior to concentration with the VivaFlow 50R system with a 10 kDa molecular weight cut-off (Sartorius). 45μL of pre-refolded HLA-E-β2M-peptide sample concentrated to 10mgs/mL, was subsequently injected onto a high-performance liquid chromatography (HPLC) KW402.5 column at Diamond Light Source Beamline B21, several hours post-concentration. HPLC elution buffers corresponded exactly to the L-Arginine Tris pH 8 macro-refolding injection buffer minus the protein components – although 60μM or 120μM excess peptide was added to the elution buffer for certain SEC-SAXS experiments. SEC-SAXS data were collected at Diamond Light Source Beamline B21 and images were taken every 3 s of X-ray-exposed HPLC-purified material over the course of a 32-min elution period. Scattering data were circularly integrated prior to buffer subtraction followed by Guinier fitting and pairwise distribution function calculations, which were performed in the SAXS-dedicated software, ScÅtter IV, developed by Robert Rambo (Diamond Light Source, 2022). Notably, the proportion of material from each protein refold eluted in the aggregate peak during the HPLC run is dependent on the affinity of the test peptide for HLA-E and is also subject to minor variations due to dynamical protein refolding equilibria. Thus, it was not possible to control the eluted, X-ray exposed protein complex concentrations during SEC-SAXS experiments. Dimensionless Kratky plots, normalised for protein concentration and mass were therefore generated, in addition to Log Intensity plots, for direct comparisons of SEC-SAXS runs. *Ab initio* molecular envelope models representing the average protein conformation in solution were also generated by the DAMMIF and DAMAVER packages of ATSAS in conjunction with ScÅtter (Franke and Svergun, 2009; Svergun, 1999; Volkov and Svergun, 2003). Superposition of such bead models to crystallographic coordinates was carried out using the Supcomb package of ATSAS (Kozin and Svergun, 2001).

#### Peptide exchange ELISA-based HLA-E peptide binding assay

Peptide exchange ELISA-based HLA-E peptide binding assays were conducted according to our previously published method that was developed and optimised by the lab (Walters et al., 2020). Peptide exchange micro-reactions were assembled in the traditional macro-refolding buffer for MHC class I molecules comprising 100 mM Tris pH8.0, 400 mM L-arginine monohydrochloride, 2 mM EDTA, 5 mM reduced glutathione, and 0.5 mM oxidized Glutathione, prepared in MiliQ water. 3 μg of previously purified HLA-E refolded with a UV-sensitive VL9 peptide variant (VMAPJTLVL) and 100 μM of “exchange” peptide were added to polypropylene V-shaped 96-well plates and final reaction volumes adjusted to 125 μL prior to 5 h incubations on ice. Although HLA-E complexes previously refolded with a UV-sensitive peptide were used, peptide exchange reactions were not irradiated in a dedicated UV cabinet since this step was previously determined unnecessary for optimal peptide exchange. For titrations experiments, the final peptide concentration of the peptide exchange reaction was varied from 7.5 μM to 1.2 mM.

Peptide exchange reactions were subsequently interrogated by sandwich ELISA (Walters et al., 2020). 96-well ELISA plates were coated in 10 mg/mL 3D12, an anti-human HLA-E capture antibody prior to a 12 h incubation period at 4°C. Following plate washing in PBS (200 μL per well) to remove excess coating antibody, ELISA plate wells were blocked with 300 μL of 2% IgG-free BSA for 2 h at RT. Blocked wells were washed five times in 0.05% Tween-based ELISA wash buffer (BioLegend) followed by a single wash in PBS prior to the addition of 50 μL of peptide exchange reaction diluted 1:100 in 2% BSA to each well. ELISA plates containing peptide exchange reaction samples were incubated for 1 h at RT and subsequently washed in 0.05% Tween-based ELISA wash buffer and PBS. A polyclonal anti-human β2M HRP-conjugated IgG detection antibody (ThermoFisher Scientific) was diluted 1:2500 in 2% BSA and 50μL added to each ELISA well. ELISA plates were incubated in the dark for 30 min prior to wash steps in a 0.05% Tween-based ELISA wash buffer and PBS. An anti-rabbit IgG enhancement antibody, raised in goats and conjugated to HRP (EnVision + System-HRP from Agilent) was diluted 1:15 in 2% BSA (containing 1% mouse serum) and subsequently added to each well. Enhancement antibodies were incubated for 15 min in ELISA wells prior to wash steps in a 0.05% Tween-based ELISA wash buffer and PBS. ELISA plates were developed in 100 μL of 3,3′,5,5′-tetramethyl benzidine (TMB) substrate (BioLegend), dark incubated at RT for 10 min and reactions terminated by the addition of 100 μL H_2_SO_4_ STOP solution (BioLegend). Absorbance readings were measured at 450 nm on a FLUOstar OMEGA plate reader and such inter-assay readings normalised via background subtraction and expression of each signal as a percentage of the positive control signal. Pearson product-moment correlation coefficients were generated for normalised ELISA signals versus various parameters obtained from SEC-SAXS analyses.

#### Differential scanning fluorimetry

Similar to previously published differential scanning fluorimetry (DSF) methodology (Anjanappa et al., 2020), the thermal stability of empty HLA-E (0.45 mg/mL) was measured following a 30 min room temperature co-incubation with 12 Molar excess of peptide (120 μM) dissolved in L-Arginine redox solution (400 mM L-Arginine monohydrochlride, 5 mM Reduced Glutathione, 0.5 mM Oxidised Glutathione, 2 mM EDTA, 100 mM Tris pH 8) in a final volume of 20 μL. Individual samples were subsequently split, transferred into two Prometheus NT.48 Series nanoDSF Grade Standard Capillaries (Nanotemper, Munich, Germany) and placed in the capillary tray of a Prometheus NT.48 fluorimeter (Nanotemper) controlled by PR.ThermControl (version 2.1.5) software. Excitation power was pre-adjusted to obtain between 8000 and 20,000 Raw Fluorescence Units for fluorescence emission at 330 nm and 350 nm. A thermal ramp ranging from 20°C to 95°C, at a rate of 1°C/min, was applied. Automated thermal melt data calling was generated by the analysis software within PR.ThermControl.

#### X-ray crystallography

100 nL of FPLC-purified peptide-refolded HLA-E at 10 mg/mL was mixed with 100 nL reservoir buffer in crystallisation wells by a Cartesian crystallisation robot and equilibrated by sitting drop vapour-diffusion at 20°C (Walter et al., 2005). Crystals of IL9 (IMYNYPAML)-bound HLA-E grew in 2.2 M ammonium sulfate, 0.1 M MES at pH 5.8 whereas Mtb14 (RMAATAQVL)-bound HLA-E crystals grew in 3 M ammonium sulfate 0.1 M MES at pH 6. Crystals were cryopreserved in 25% glycerol and diffraction data were collected at the Diamond Light Source, beamlines i04 (HLA-E-Mtb14 structure) and i03 (HLA-E-IL9 structure). Diffraction data were auto-indexed by Xia2 DIALS. Since the outer shell CC_1/2_ exceeded the minimum threshold (>0.5) for both datasets, no reflections were excluded from downstream analysis. A more conservative data truncation approach according to the R_merge_ and I/sigma cut-offs has been shown to result in the elimination of useful data which would otherwise have contributed to model quality (Karplus and Diederichs, 2012). Thus, the well-established and reproducible indicator of crystallographic data quality, CC_1/2_, superseded such traditional thresholds and was adopted as a determinant for data truncation for the crystal structures presented in this study. Despite anisotropic X-ray diffraction for HLA-E-Mtb14, anisotropy correction resulted in unacceptably large reductions in dataset completeness (<90%), and thus original diffraction data were used for downstream processing. Molecular replacement was conducted by MolRep of the CCP4i suite using published crystallographic coordinates of HLA-E (PDB ID: 6GH1), with the Mtb44 (RLPAKAPLL) peptide coordinates removed, as an initial phasing model (Murshudov et al., 2011; Vagin and Teplyakov, 2010; Walters et al., 2018; Winn et al., 2011). Rigid body, restrained and TLS refinement were computed by the CCP4i REFMAC5 (Murshudov et al., 2011) or Phenix.refine (Afonine et al., 2012) between iterative cycles of manual model building in Coot (Emsley et al., 2010). Geometry was validated by MolProbity (Chen et al., 2010), prior to visualisation of the model in the PyMOL Molecular Graphics System, version 2.0 (Schrödinger, LLC) and further investigation using PDBePISA) (Krissinel and Henrick, 2007) and PDBeFOLD (Krissinel and Henrick, 2004). Data collection and refinement statistics are detailed in Table S2.

### Quantification and statistical analysis

Pearson product-moment correlation coefficients were calculated for DSF-determined melting temperatures and normalised ELISA-based peptide binding signals, and linear correlations were identified between melting temperatures or normalised ELISA-based signals and various SEC-SAXS parameters including the maximal dimensions of the average conformation in solution (d_max_) or the volume of the DAMMIF *ab initio* molecular envelope model (Å^3^). Data were analyzed using Prism 7 (GraphPad Software). Statistical significance was determined using either an unpaired or paired t test. Plots show mean ± SEM. (^∗^p < 0.05, ^∗∗^p < 0.01, ^∗∗∗^p < 0.001, ^∗∗∗∗^p < 0.0001).

## Data Availability

• The HLA-E^∗^01:03^IL9^and HLA-E^∗^01:03^Mtb14^ crystal structures have been deposited in the Protein DataBank (https://www.rcsb.org/structure/) under PDB accession codes 7P4B and 7P49, respectively. • This paper does not report original code. • Any additional information required to re-analyze the data reported in this paper is available from the lead contact upon request.

## Supplemental information

Document S1. Figures S1–S5 and Tables S1–S3

Document S2. Article plus supplemental information
