# Supplementary material for: Primary and secondary functions of HLA-E are determined by stability and conformation of the peptide-bound complexes
Source: Cell Rep. 2022 Jun 14;39(11):110959. doi: 10.1016/j.celrep.2022.110959 (PMC9380258; doi:10.1016/j.celrep.2022.110959)
Supplement: Document S1. Figures S1–S5 and Tables S1–S3 [file mmc1.pdf]

Cell Reports, Volume *39*

## Supplemental information

# Primary and secondary functions of HLA-E are determined by stability and conformation of the peptide-bound complexes

**Lucy C. Walters, Daniel Rozbesky, Karl Harlos, Max Quastel, Hong Sun, Sebastian Springer, Robert P. Rambo, Fiyaz Mohammed, E. Yvonne Jones, Andrew J. McMichael, and Geraldine M. Gillespie**

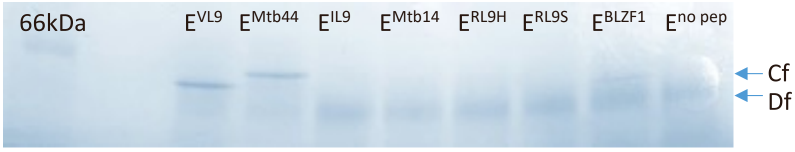


**Supplementary Figure 1. Distinct blue native gel signatures for pathogen peptide versus canonical VL9 leader peptide-loaded HLA-E complexes. Related to Figure 1.**

10μg of pre-refolded HLA-E-β2m-UV-sensitive VL9 (VMAPJTVLL) material was incubated with 12M excess of test peptides (VL9, Mtb44, IL9, RL9H, RL9S and BZLF1) or without added peptide ($E^{no\ pep}$) for 3 hours on ice prior to Blue Native-PAGE™ Novex 4-16% Bis-Tris gel evaluation. Gel electrophoresis was performed at 150 volts for 2 hours at room temperature (RT), over a current dropping from 15-16 down to 2-4mAmps. Gels were subsequently rinsed in MilliQ water and stained for 2-3 hours, at RT, in SimplyBlue™ SafeStain. Final de-staining steps were performed in MilliQ water over a period of 24 hours prior to imaging. The NativeMark™ protein standard 66kDa band is denoted for reference. The blue arrows denote both Compact (Cf) and Diffuse (Df) HLA-E-peptide loaded gel forms.

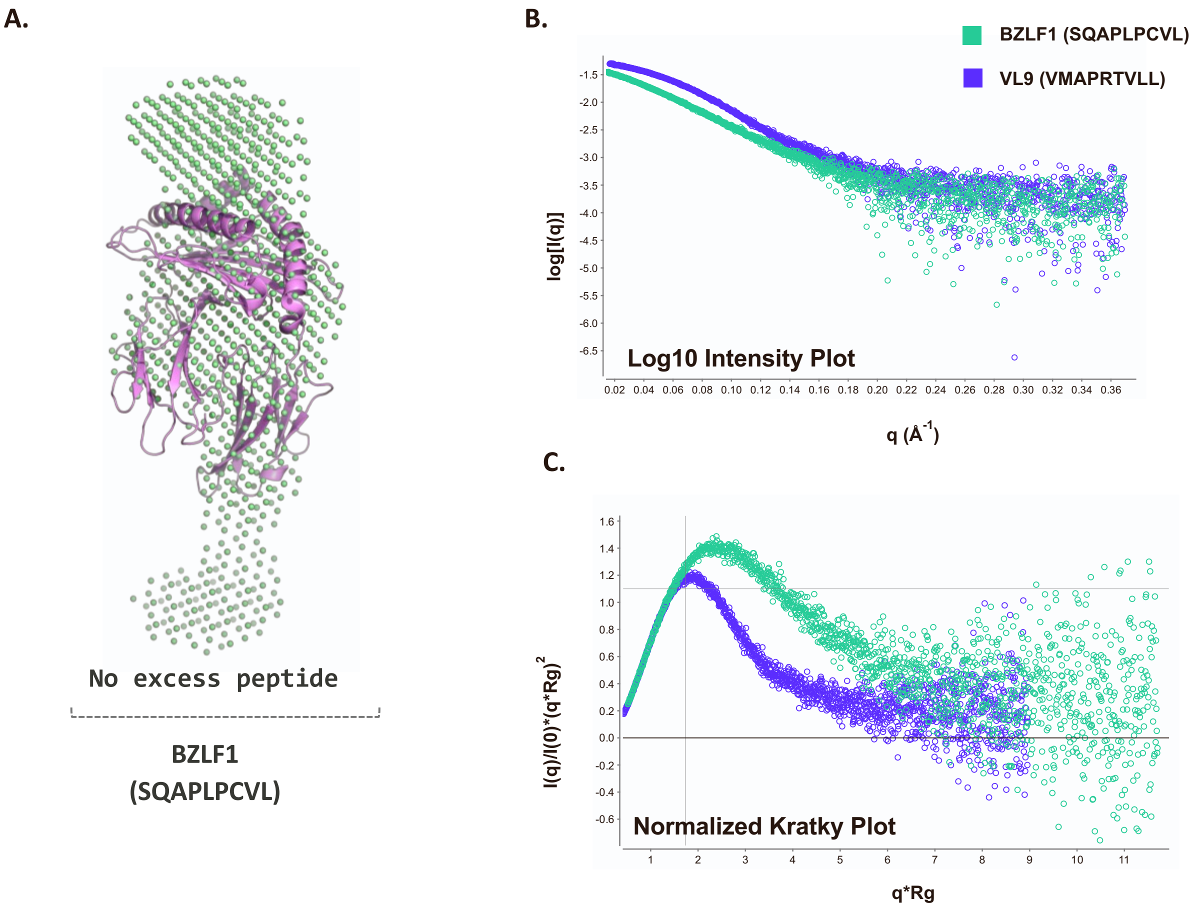


D.

| Peptide ID | Sequence | Organism | $R_g$ (Å) | $d_{max}$ (Å) | Molecular envelope Vol (Å³) | Reference |
|---|---|---|---|---|---|---|
| BZLF1 | SQAPLPCVL | EBV | 34.5 | 108 | 107,950 | Jørgensen, P,B. et al. (2012) |

**Supplementary Figure 2. Size exclusion chromatography-coupled small angle x-ray scattering for HLA-E refolded with the EBV-derived peptide, BZLF1 (SQAPLPCVL). Related to Figure 1.**

A. *Ab initio* DAMMIF model (small green 'dots') representing the average conformational state of HLA-E refolded with the EBV-derived peptide, BZLF1 (SQAPLPCVL), generated from SEC-SAXS data using the DAMMIF and DAMAVER ATSAS packages with ScÅtter (Franke & Svergun, 2009) (Volkov & Svergun, 2003). The HLA-E*01:03 refold was injected onto the HPLC column in the presence of 120µM excess BZLF1 peptide but without excess peptide in the HPLC elution buffer. In the absence of a of HLA-E-BZLF1 crystal structure, the structural coordinates for HLA-E-RL9HIV (RMYSPTSIL) (PDB: 6GL1) was aligned to the DAMMIF model, using the SUPCOMB package of ATSAS, for reference (Walters et al, 2018). B. Log10 scattering intensity plot for HLA-E SEC-SAXS. The scattering vector, q, in Å-1, which for small angles is proportional to the scattering angle θ is plotted on the x-axis versus the scattered intensity, I(q) on the y-axis (log scale). Scattering intensity curves for HLA-E refolds run in the absence of excess peptide in the HPLC elution buffer are plotted for HLA-E-BZLF1 (SQAPLPCVL) and HLA-E-VL9 (VMAPRTVLL) and are colour-coded according to the corresponding figure legend. (ii) Normalised Kratky plot with superimposed curves corresponding to SEC-SAXS experiments for HLA-E-BZLF1 (SQAPLPCVL) and HLA-E-VL9 (VMAPRTVLL) run in the absence excess peptide in the HPLC elution buffer, colour-coded according to the figure legend in C. Plotted on the x-axis is the scattering vector multiplied by the radius of gyration. On the y-axis the scattering intensity I(q) is divided by the experiment's I(0) and multiplied by (q*Rg)2. The x- and y-axis units are chosen such that the peak of the modulated Gaussian curve will always lie at q*Rg = √3 with a magnitude of 3·e-1, regardless of protein size and concentration when the Guinier's approximation is obeyed. A shift from this peak indicates an increased degree of protein flexibility or conformational asymmetry. D. Table detailing the HLA-E-BZLF1 (SQAPLPCVL) refold tested via SEC-SAXS where 120µM excess peptide was present in the HPLC injection buffer and where no excess peptide was included in the HPLC elution buffer. The radius of gyration (Rg) and maximum dimension (dmax), both in Å, and volume of the SEC-SAXS 'dot' model (Å$^3$) displayed in part A, are denoted.

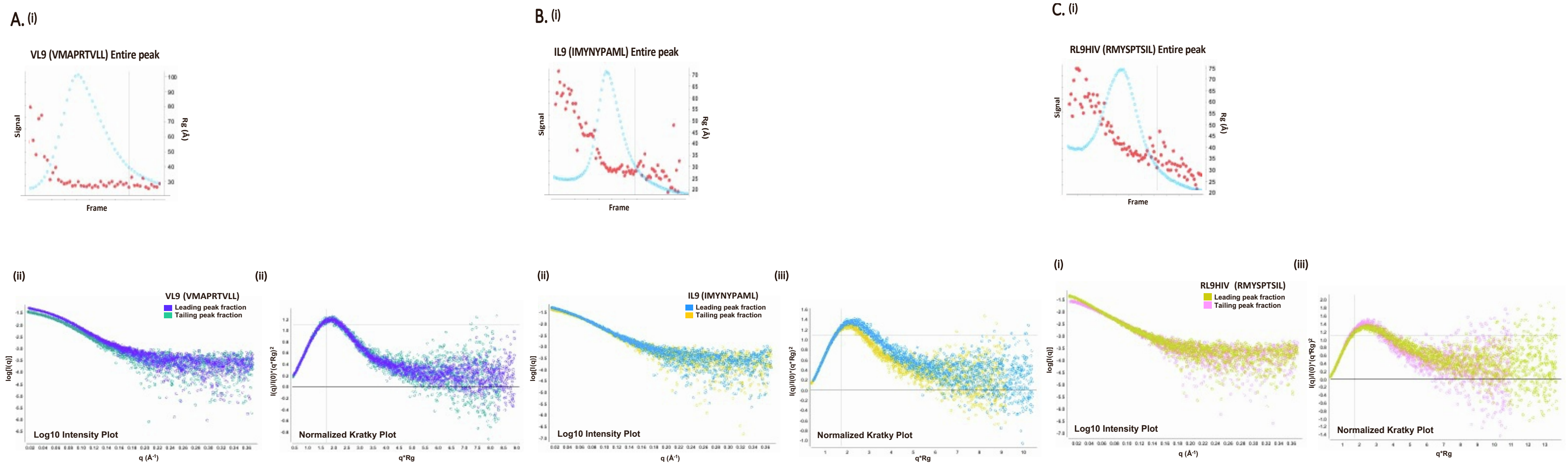


(iv)

| Peptide ID | Sequence | Organism | Peak Fraction | $R_g$ (Å) | $d_{max}$ (Å) | Reference |
|---|---|---|---|---|---|---|
| VL9 | VMAPRTVLL | Human | Leading | 26.0 | 96 | O'Callaghan et al. (1998) |
| | | | Tailing | 25.1 | 80 | |

(iv)

| Peptide ID | Sequence | Organism | Peak Fraction | $R_g$ (Å) | $d_{max}$ (Å) | Reference |
|---|---|---|---|---|---|---|
| IL9 | IMYNYPAML | Mtb | Leading | 32.4 | 113 | McMurtrey, C. et al. (2017) |
| | | | Tailing | 28.5 | 100 | |

(iv)

| Peptide ID | Sequence | Organism | Peak Fraction | $R_g$ (Å) | $d_{max}$ (Å) | Reference |
|---|---|---|---|---|---|---|
| RL9HIV | RMYSPTSIL | HIV | Leading | 44.2 | 152 | Walters, L.C, et al (2018) |
| | | | Tailing | 34.4 | 109 | |

**Supplementary Figure 3. Heterogeneous conformational ensembles of pathogen peptide-refolded HLA-E. Related to Figure 1**

A(i)-C(i). Graphs showing the radius of gyration ($R_g$) in Å, denoted as red dots plotted across SAXS curves, corresponding to the elution curves for VL9 (VMAPRTVLL), IL9 (IMYNYPAML) and RL9HIV (RMYSPTSIL) refolded HLA-E. The x-axis denotes HPLC-eluted x-ray exposed frames across the elution frames across the HLA-E refolded peaks and the y-axis corresponds to the SAXS scatter intensity signals.
A(ii)-C(ii). Log10 scattering intensity plot for HLA-E SEC-SAXS. On the x-axis is the scattering vector, q, measured in Å-1 (which for small angles is proportional to the scattering angle, θ) versus the scattered intensity I(q), on the y-axis (log-scale). Scattering intensity curves for VL9, IL9 and RL9HIV-refolded HLA-E run in the absence of 120 μM excess peptide in the HPLC elution buffer are plotted for the leading and tailing peak factions of HLA-E complexes.
A(iii)-C(iii). Normalised Kratky plots with superimposed curves corresponding to SEC-SAXS leading and tailing peaks of HLA-E-peptide complexes in the absence of 120 μM excess peptide in the HPLC elution buffer. Plotted on the x-axis is the scattering vector multiplied by the radius of gyration. On the y-axis, the scattering intensity I(q) is divided by the experiment's I(0) and multiplied by (q*Rg)2. The units on the x- and y-axes are chosen such that the peak of the modulated Gaussian curve will always lie at q*Rg = √3 with a magnitude 3.e-1, regardless of protein size and concentration, when Guinier's approximation is obeyed – true for globular and compact proteins. A shift from this peak signifies an increased degree of protein flexibility or conformational asymmetry.

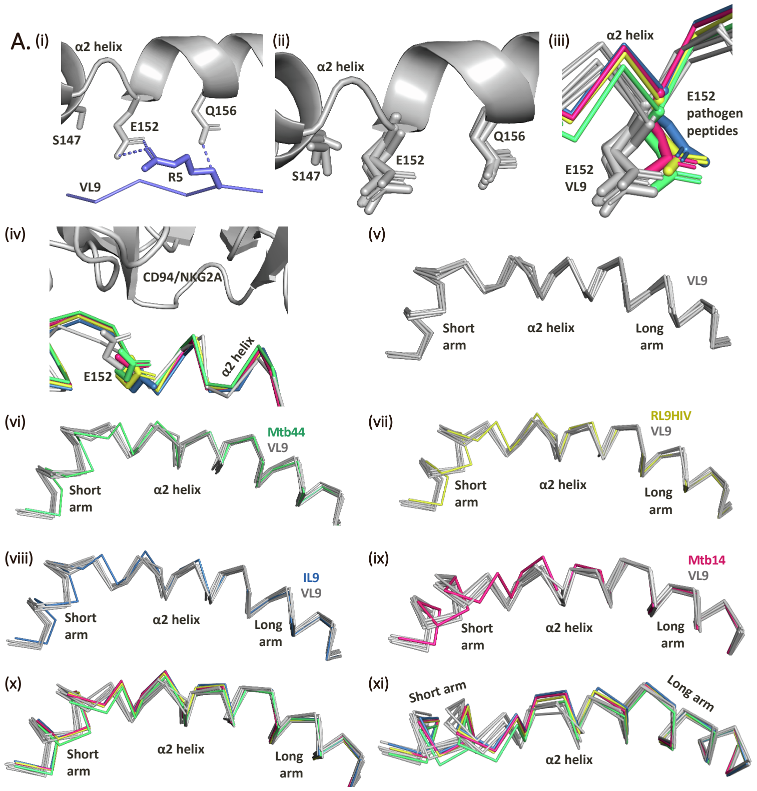


(xii)

| Peptide ID | Origin | Sequence | Allele | PDB AC | Ref |
|---|---|---|---|---|---|
| | HLA-B7 | VMAPRTVLL | HLA-E*01:01 | 1MHE | O'Callaghan et al. 1998 |
| | | VMAPRTVLL | HLA-E*01:03 | 1KPR | Holmes et al. 2003 |
| VL9 Leader Peptide | HLA-B27 | VTAPRTLLL | HLA-E*01:03 | 1KTL | Holmes et al. 2003 |
| | HLA-Cw*07 | VMAPRALLL | HLA-E*01:01 | 3BZF | Hoare et al. 2008 |
| | HLA-G | VMAPRTLFL | HLA-E*01:01 | 3BZE | Hoare et al. 2008 |
| VL9 'mimic' peptide | CMV | VMGPRTLIL | HLA-E*01:01 | 3AM8 | Chacko et al. 2011 |
| Mtb44 | Mtb | RLPAKAPLL | HLA-E*01:03 | 6GH1 | Walters et al. 2018 |
| RL9HIV | HIV | RMYSPTSIL | HLA-E*01:03 | 6GL1 | Walters et al. 2018 |
| IL9 | Mtb | IMYNYPAML | HLA-E*01:03 | ** | Present study |
| Mtb14 | Mtb | RMAATAQVL | HLA-E*01:03 | ** | Present study |

**Supplementary Figure 4. Differential α2 helical kink configurations distinguish leader peptide-associated versus pathogen epitope-bound HLA-E. Related to Figure 6**

A. (i) PyMol visualisation of the HLA-E α2 helix (grey cartoon) and VL9 (VMAPRTVLL) (purple ribbon) from published coordinates, PBD: 1MHE. VL9 position 5 Arg and HLA-E α2 helix residues Ser-147, Glu-152 and Gln-156 side chains are displayed (stick-form), with inter-chain polar contacts (purple dashed lines). (ii) 6 published HLA-E structures in complex with VL9 variant peptides (PDB: 1MHE, PDB: 1KPR, PDB: 1KTL, PDB: 3BZF, PDB: 3BZE and PDB: 3AM8) are superimposed, with the α2 helix (grey cartoon) and residues Ser-147, Glu-152 and Gln-156 (grey sticks) displayed. (iii) 6 published HLA-E-VL9 structures (described in (ii), grey) plus 4 HLA-E structures - HLA-E-Mtb44 (RLPAKAPLL) in green, HLA-E-IL9 (IMYNYPAML) in blue, HLA-E-Mtb14 (RMAATAQVL) in magenta and HLA-E-RL9HIV (RMYSPTSIL) in yellow - are superimposed, with the α2 helix (ribbon-form) and the Glu-152 side chain (stick-form) denoted. (iv) The 4 HLA-E pathogen-peptides complexes from (iii) are superimposed to an HLA-E-VL9 (VMAPRTLFL) molecule (grey) from the published HLA-E-CD94/NKG2A co-complex structure, PDB: 3CDG. The CD94/NKG2A receptor (grey cartoon), the superimposed HLA-E α2 helices (ribbons) and the Glu-152 side chain (sticks) are denoted. (v) The α2 helices from 6 superimposed HLA-E-VL9 structures (described in (ii)) are depicted (grey ribbons) alone or superimposed to (vi) the Mtb44 peptide (green), (vii) the RL9HIV peptide (yellow), (viii) the IL9 peptide (IMYNYPAML) (blue) and (ix) the Mtb-derived Mtb14 peptide (magenta). (x) The α2 helices from 10 superimposed HLA-E structures are viewed side-on, including 6 HLA-E-VL9 complexes (described in (ii), grey) and 4 in complex with pathogen-derived peptides, colour-coded according to A. (xi) Aerial view of A. (xii) Table summarising the 6 HLA-E-VL9 (described in (ii)) plus 4 HLA-E structures in complex with pathogen-derived peptides, 2 reported here. Peptide ID, origin and sequence, PDB accession code and further details are reported.

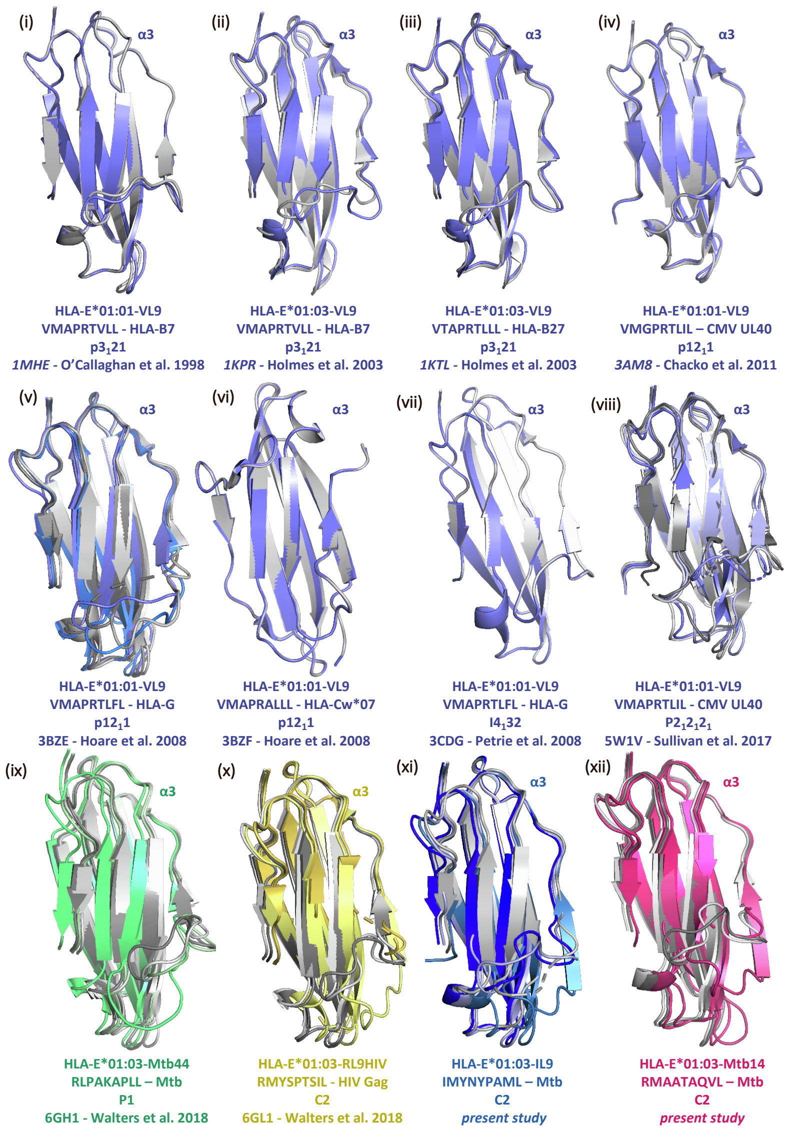


**Supplementary Figure 5. HLA-E α3 domain superposition, Related to Figure 6**

A. PyMol visualisation of HLA-E α3 domains following alignment of HLA-E-β2M-peptide complexes present within the asymmetric unit of the previously published HLA-E*01:01-VL9 (VMAPRTVLL) structure, PDB: 1MHE (i), the previously published HLA-E*01:03-VL9 (VMAPRTVLL) structure, PDB: 1KPR (ii), the previously published HLA-E*01:03-VL9 (VTAPRTLLL) structure, PDB: 1KTL (iii), the previously published HLA-E*01:01-VL9 (VMGPRTLIL) structure, PDB: 3AM8 (iv), the previously published HLA-E*01:01-VL9(VMAPRTLFL) structure, PDB: 3BZE (v) and the previously published HLA-E*01:01-VL9 (VMAPRALLL) structure, PDB: 3BZF (vi). (vii) PyMol visualisation of HLA-E α3 domains following alignment of HLA-E-β2M-peptide complexes from the asymmetric unit of the previously published CD94/NKG2A-HLA-E*01:01-VL9 (VMAPRTLFL) co-complex crystal structure, PDB: 3CDG. (viii) PyMol visualisation of HLA-E α3 domains following alignment of HLA-E-β2M-peptide complexes from the asymmetric unit of the previously published TCR-HLA-E*01:01-VL9 (VMAPRTLIL) co-complex crystal structure, PDB: 5W1V. PyMol visualisation of HLA-E α3 domains following alignment of HLA-E-β2M-peptide complexes present within the asymmetric unit of the previously published HLA-E*01:03-Mtb44 (RLPAKAPLL) structure, PDB: 6GH1 **(ix)**, the previously published HLA-E*01:03-RL9HIV (RMYSPTSIL) structure, PDB: 6GL1 **(x),** and the HLA-E*01:03-IL9 (IMYNYPAML) **(xi)** and HLA-E*01:03-Mtb14 (RMAATAQVL) **(xii)** structures presented in this study.

| Peptide ID | Sequence | Origin | ELISA Rank (%VL9 binding) | $T_m$ (°C) 12M | $d_{max}$ (Å) | Molecular envelope Vol (Å$^3$) | Reference |
|---|---|---|---|---|---|---|---|
| **VL9** | **VMAPRTVLL** | **Human, HLA-B7/8/14/39/42/48** | **100** | **49.4** | **87** | **78,628** | **O'Callaghan et al. (1998)** |
| **Mtb44** | **RLPAKAPLL** | ***Mtb,* Rv1484** | **97** | **50.6** | **97** | **84,171** | **Joosten, S.A, et al. (2010)** |
| **IL9** | **IMYNYPAML** | ***Mtb,* EsxH** | **65** | **40.7** | **93** | **85348** | **McMurtrey, C. et al. (2017)** |
| **Mtb14** | **RMAATAQVL** | ***Mtb,* Rv2932** | **48** | **37.8** | **120** | **123,647** | **Joosten, S.A, et al. (2010)** |
| **RL9HIV** | **RMYSPTSIL** | ***HIV,* Gag** | **43** | **38.2** | **127** | **146,874** | **Walters, L.C, et al (2018)** |
| **BZLF1** | **SQAPLPCVL** | ***EBV,* BZLF1** | **41** | **37.7** | **108** | **107,950** | **Jørgensen, P,B. et al. (2012)** |
| **RL9SIV** | **RMYNPTNIL** | ***SIV,* Gag** | **22** | **35.2** | **155** | **161,392** | **Hansen, S,G. et al (2016)** |
| **Neg Ctrl pep** | **QAISPRTLN** | ***HIV,* Gag** | **0.3** | **31.8** | **-** | **-** | **Walters, L.C, et al. (2020)** |
| **'Empty'** | **No peptide** | **-** | **0** | **32.0** | **-** | **-** | **-** |

**Supplementary Table 1. Relationship between ELISA-based peptide binding signals, thermal stability measurements and size exclusion chromatography-coupled small angle x-ray scattering (SEC-SAXS) data. Related to Figure 1**

The biological origin and corresponding references for each pathogen-derived HLA-E-restricted peptides are listed. A positive control VL9 leader peptide (VMAPRTVLL) in addition to a non-binding negative control peptide derived from HIV Gag (QAISPRTLN) are included. The 'ELISA rank' column reflects normalised, previously published peptide binding signals which are expressed as percentages of the positive control VL9 signal (Walters et al. 2020). HLA-E-β2M-peptide complex thermal stability is indicated by its melting temperature (°C) in the presence of 12M excess peptide. The SEC-SAXS-obtained $d_{max}$ value, measured in Å, reflects the maximum dimension across the HLA-E complex in solution whereas the 'Molecular Envelope Vol' corresponds to the volume in Å$^3$ of the DAMMINF *ab initio* molecular envelope models presented in Figure 2.

| Data collection | HLA-E*01:03 in complex with peptide: IL9 (IMYNYPAML) | Mtb14 (RMAATAQVL) |
|---|---|---|
| Crystallisation condition | 2.2 M AS<br>0.1 M MES<br>pH 5.8 | 3 M AS<br>0.1 M MES<br>pH 6 |
| Resolution Å | 114.03-1.72<br>(1.75-1.72) | 57.16-2.05<br>(2.124-2.05) |
| Space Group | C2 | C2 |
| Cell dimensions | a =244.56<br>b = 48.53<br>c = 153.16<br>α = 90.0°<br>β = 116.95°<br>γ = 90.0° | a = 245.15 Å<br>b = 47.93 Å<br>c = 152.45 Å<br>α = 90°<br>β = 117.45°<br>γ = 90° |
| Solvent content [%]<br>(molecules per AU) | 45 (4) | 44 (4) |
| Unique reflections | 171397 (8228) | 99611 (9754) |
| Completeness [%] | 99.8 (96.6) | 98.95 (96.24) |
| $R_{merge}$ [I] | 0.12 (1.88) | 0.155 (0.828) |
| I/sigma | 8.1 (1.0) | 9.7 (1.8) |
| Multiplicity | 6.5 (5.5) | 12.7 (8.2) |
| $CC_{1/2}$ | 1.0 (0.56) | 0.99 (0.60) |
| **Refinement** | | |
| No. of non-hydrogen atoms | 13404 | 13201 |
| $R_{factor}$ [%)] | 0.215 (0.332) | 0.2305 |
| $R_{free}$ [%] | 0.241 (0.348) | 0.2730 |
| r.m.s.d. bonds [Å] § | 0.004 | 0.008 |
| r.m.s.d. angles [deg] | 0.75 | 1.008 |
| Ramachandran statistics: | | |
| Favoured [%] | 98.7 | 98.72 |
| Disallowed [%] | 0.2 | 0 |

**Supplementary Table 2. Crystallographic data collection and refinement statistics for HLA-E*01:03 structures with the *Mycobacterial* peptides, IL9 (IMYNYPAML) and Mtb14 (RMAATAQVL). Related to Figure 4.**

AS: Ammonium sulphate. § r.m.s.d.: root mean squared deviation from ideal geometry. Statistics for the highest resolution shell are shown in parentheses. AU: asymmetric unit. Rfree equals the R factor against 5% of the data removed prior to refinement.

| Species | HLA Allele | P147 | P152 | P156 |
|---|---|---|---|---|
| Human | HLA-E*01:01 | Ser | Glu | Gln |
| | HLA-E*01:03 | Ser | Glu | Gln |
| Mouse | Qa-1a | Ser | Glu | Gln |
| | Qa-1b | Ser | Glu | Gln |
| | Qa-1c | Ser | Glu | Gln |
| | Qa-1d | Ser | Glu | Gln |
| Rhesus macaque | Mamu-E*02:01:02 | Ser | Glu | Gln |
| | Mamu-E*02:02 | Ser | Glu | Gln |
| | Mamu-E*02:03 | Ser | Glu | Gln |
| | Mamu-E*02:04 | Ser | Glu | Gln |
| | Mamu-E*02:05 | Ser | Glu | Gln |
| | Mamu-E*02:07 | Ser | Glu | Gln |
| | Mamu-E*02:09 | Ser | Glu | Gln |
| | Mamu-E*02:10 | Ser | Glu | Gln |
| | Mamu-E*02:11 | Ser | Glu | Gln |
| | Mamu-E*0212:01 | Ser | Glu | Gln |
| | Mamu-E*0212:02 | Ser | Glu | Gln |
| | Mamu-E*02:13 | Ser | Glu | Gln |
| | Mamu-E*02:14 | Ser | Glu | Gln |
| | Mamu-E*02:15 | Ser | Glu | Gln |
| | Mamu-E*02:16 | Ser | Glu | Gln |
| | Mamu-E*02:17 | Ser | Glu | Gln |
| | Mamu-E*02:18 | Ser | Glu | Gln |
| | Mamu-E*02:19 | Ser | Glu | Gln |
| | Mamu-E*02:20 | Ser | Glu | Gln |
| | Mamu-E*02:21 | Ser | Glu | Gln |
| | Mamu-E*02:22 | Ser | Glu | Gln |
| | Mamu-E*02:23 | Ser | Glu | Gln |
| Human | HLA-A*68:01 | Trp | Val | Trp |
| | HLA-A*30:03 | Trp | Arg | Leu |
| | HLA-A*02:01 | Trp | Val | Leu |
| | HLA-A*24:02 | Trp | Val | Gln |
| | HLA-A*11:01 | Trp | Ala | Gln |
| | HLA-A*02:06 | Trp | Val | Leu |
| | HLA-A*0301 | Trp | Glu | Leu |
| | HLA-A*02:03 | Trp | Glu | Trp |
| | HLA-A*02:07 | Trp | Val | Leu |
| | HLA-A*01:01 | Trp | Ala | Arg |
| | HLA-B*44:05 | Trp | Val | Asp |
| | HLA-B*37:01 | Trp | Val | Asp |
| | HLA-B*18:01 | Trp | Val | Leu |
| | HLA-B*14:02 | Trp | Glu | Leu |
| | HLA-B*44:02 | Trp | Val | Asp |
| | HLA-B*44:03 | Trp | Val | Leu |
| | HLA-B*27:05 | Trp | Val | Leu |
| | HLA-B*27:03 | Trp | Val | Leu |
| | HLA-B*35:05 | Trp | Val | Arg |
| | HLA-B*08:01 | Trp | Val | Asp |
| | HLA-B*57:03 | Trp | Val | Leu |
| | HLA-C*06:02 | Trp | Glu | Trp |
| | HLA-C*0801 | Trp | Thr | Leu |
| | HLA-C*05:01 | Trp | Glu | Arg |

**Supplementary Table 3. Sequence conservation among human, murine and rhesus macaque MHC-E alleles of differentially positioned $\alpha$2 helix residues. Related to Figure 6.**

Table detailing amino acid residues at positions 147, 152 and 156 of the $\alpha$2 helix of human, murine and rhesus macaque MHC-E alleles – such residues were differentially positioned in HLA-E structures in complex with pathogen peptide versus the canonical VL9 leader sequence peptide. A random selection of 24 classical MHC class I molecules were included for sequence comparison.
